# The crystal structure of a mononuclear Pr^III^ complex with cucurbit[6]uril

**DOI:** 10.1107/S2056989024005760

**Published:** 2024-06-25

**Authors:** George V. Fedorenko, Oleksandr I. Zbruyev, Anna V. Pavlishchuk, Lyudmila P. Oleksenko, Sergiu G. Shova, Valentyn A. Chebanov, Vitaly V. Pavlishchuk

**Affiliations:** a L. V. Pisarzhevskii Institute of Physical Chemistry of the National Academy of Sciences of Ukraine, Prospect Nauki 31, Kyiv, 03028, Ukraine; b SSI "Institute for Single Crystals" of National Academy of Sciences of Ukraine, 60 Nauky ave., Kharkiv 61072, Ukraine; c Department of Chemistry, Purdue University, 560 Oval Drive, West Lafayette, 47907-2084, IN, USA; d Department of Chemistry, Taras Shevchenko National University of Kyiv, Volodymyrska str. 62, Kyiv, 01601, Ukraine; e Department of Inorganic Polymers, Petru Poni Institute of Macromolecular Chemistry, Aleea Grigore Ghica Voda nr. 41A, Iaşi, 700487, Romania; f V. N. Karazin Kharkiv National University, 4 Svobody sq., Kharkiv 61077, Ukraine; Universidade de Sâo Paulo, Brazil

**Keywords:** crystal structure, lanthanide(III), mononuclear complex, cucurbit[6]uril

## Abstract

A new mononuclear complex, penta­aqua­(cucurbit[6]uril-κ^2^*O*,*O*′)(nitrato-κ^2^*O*,*O*′)praseodymium(III) dinitrate 9.56-hydrate, [Pr(NO_3_)(CB6)(H_2_O)_5_](NO_3_)_2_·9.56H_2_O (**1**), was obtained as outcome of the hydro­thermal reaction between the macrocyclic ligand cucurbit[6]uril (**CB6**, C_36_H_36_N_24_O_12_) with a tenfold excess of Pr(NO_3_)_3_·6H_2_O. Complex **1** crystallizes in the *P*2_1_/*n* space group with two crystallographically independent but chemically identical [Pr(CB6)(NO_3_)(H_2_O)_5_]^2+^ complex cations, four nitrate counter-anions and 19.12 inter­stitial water mol­ecules per asymmetric unit. The nona­coordinated Pr^III^ in **1** are located in the PrO_9_ coordination environment formed by two carbonyl O atoms from bidentate cucurbit[6]uril units, two oxygen atoms from the bidentate nitrate anion and five water mol­ecules. Considering the differences in Pr—O bond distances and O—Pr—O angles in the coordination spheres, the coordination polyhedrons of the two Pr^III^ atoms can be described as distorted spherical capped square anti­prismatic and muffin polyhedral.

## Abstract 2

A reaction between cucurbit[6]uril (**CB6**) with an excess of Pr(NO_3_)_3_·6H_2_O lead to the isolation of new mononuclear complex [Pr(CB6)(NO_3_)(H_2_O)_5_](NO_3_)_2_·9.56H_2_O (**1**), which crystallizes in the *P*2_1_/*n* space group. The asymmetric unit of **1** contains two crystallographically independent [Pr(CB6)(NO_3_)(H_2_O)_5_]^2+^ complex cations, four nitrate counter-anions for charge balance and 19.12 inter­stitial water mol­ecules. The coordination environments of the Pr^III^ ions in **1** are formed by two carbonyl O atoms from bidentate cucurbit[6]uril units, two oxygen atoms from the bidentate nitrate anion and five water mol­ecules.

## 1. Chemical context

Cucurbit[*n*]urils (CB[*n*]s) are 3D cyclic organic mol­ecules possessing a rigid hydro­phobic macrocyclic cavity, which is available for the uptake of various guest mol­ecules *via* non-covalent inter­actions (Lin *et al.*, 2020 ▸). Recently, the main inter­est in cucurbit[*n*]uril chemistry was due to their possible applications in selective catalysis (Nandi *et al.*, 2017 ▸), mol­ecular recognition (Barrow *et al.*, 2015 ▸) and drug delivery (Das *et al.*, 2019 ▸). The presence of several carbonyl oxygen atoms on both sides of the macrocyclic ring makes cucurbit[*n*]urils attractive ligand platforms for the design of discrete and polymeric coordination compounds, which can provide accessible channels due to the peculiarities of the arrangement of the cucurbit[*n*]urils in the crystal structure (Ni *et al.*, 2013 ▸). The design of lanthanide(III) complexes with cucurbit[*n*]urils is particularly inter­esting because of the possible applications in mol­ecular magnetism (Ren *et al.*, 2013 ▸) and luminescence (Matsumoto *et al.*, 2022 ▸). Depending on the size of the macrocyclic cavity and the lanthanide(III) ionic radii, cucurbit[*n*]urils usually provide two to six oxygen atoms in the coord­ination sphere of the lanthanide ions (Zhang *et al.*, 2019 ▸, 2020 ▸; Liang *et al.*, 2013*b* ▸; Zheng & Liu, 2017 ▸). In the majority of cases, the inter­action between cucurbit[*n*]urils and lanthan­ide(III) salts leads to the formation of discrete mononuclear assemblies with one coordinated cucurbit[*n*]uril (Ren *et al.*, 2013 ▸; Ni *et al.*, 2015 ▸); however, examples of polynuclear complexes and coordination polymers have also been reported (Zhang *et al.*, 2019 ▸; Zhang *et al.*, 2020 ▸; Liang *et al.*, 2013*a* ▸,*b* ▸). Several lanthanide-containing complexes with cucurbit[6]urils have been reported previously (Ren *et al.*, 2013 ▸; Zheng & Liu, 2017 ▸; Shan *et al.*, 2016 ▸; Yang *et al.*, 2016 ▸; Xiao *et al.*, 2016 ▸). In the absence of additional bridging organic ligands or anionic complexes, the inter­action between *Ln*^III^ salts and cucurbit[6]uril leads to the formation of mononuclear complexes (Ren *et al.*, 2013 ▸; Yang *et al.*, 2016 ▸; Kovalenko *et al.*, 2021 ▸; Samsonenko *et al.*, 2002 ▸).
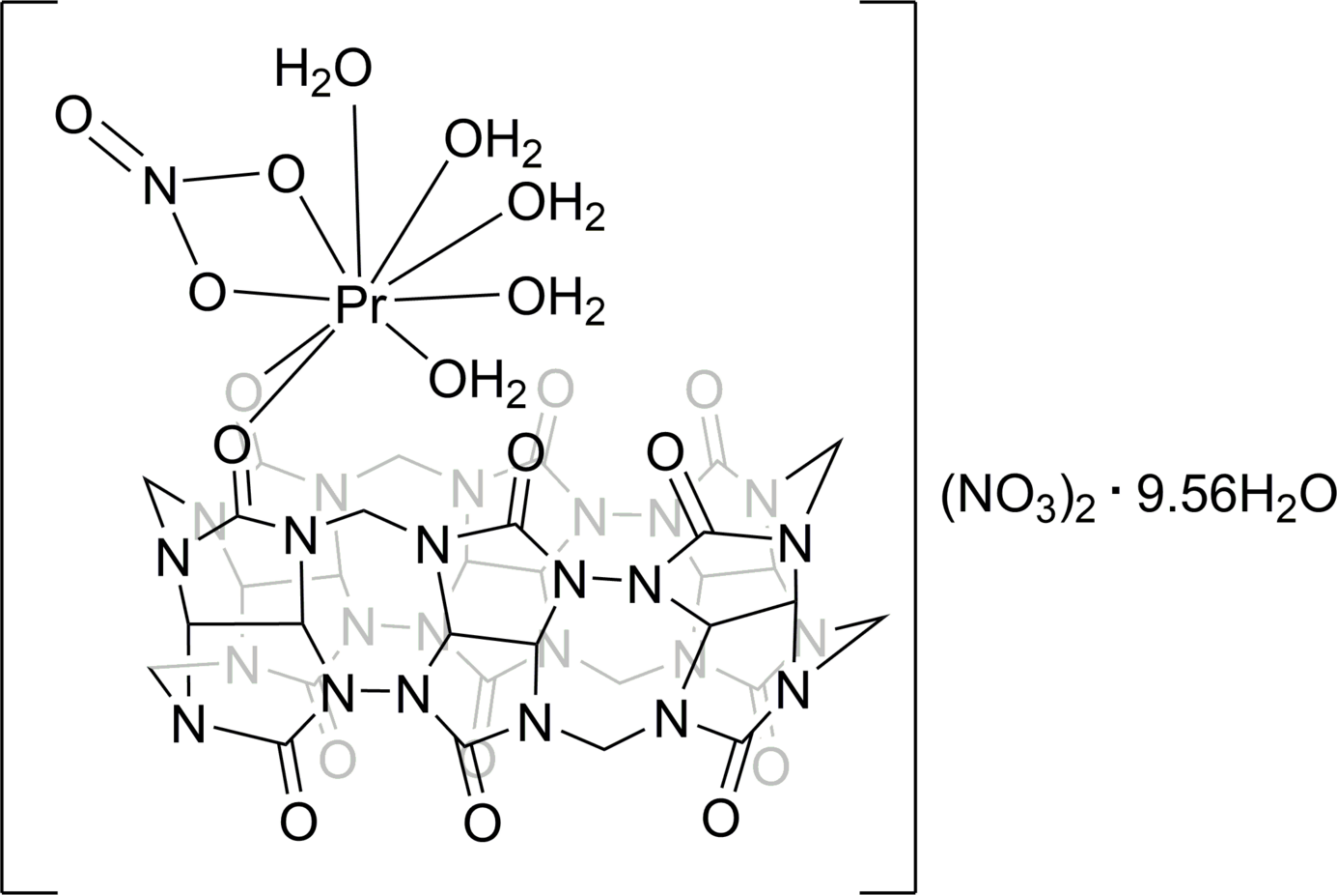


Since cucurbit[6]uril is poorly soluble in water, lanthanide complex formation is usually observed in the presence of strong mineral acids (da Silva *et al.*, 2014 ▸) or a substantial excess of the lanthanide salt reaching 25-fold excess (Ren *et al.*, 2013 ▸). The observed structure of lanthanide(III)–cucurbit[6]uril complexes depends upon a number of factors, which include an excess of the lanthanide salt in the reaction mixture, the counter-anion, reaction temperature and crystallization conditions.

In this work we report synthesis and crystal structure of a new mononuclear Pr^III^ complex with cucurbit[6]uril, [Pr(**CB6**)(NO_3_)(H_2_O)_5_](NO_3_)_2_·9.56 H_2_O (**1**), which was synthesized in the presence of a lowered tenfold Pr^III^ excess and is not isomorphous to previously reported *Ln*^III^ complexes with cucurbit[6]uril.

## 2. Structural commentary

The title complex **1** was prepared and isolated as colorless crystals according to a modified procedure for the analoguous Dy^III^ complex with cucurbit[6]uril, using Pr(NO_3_)_3_·6H_2_O for **1** (Ren *et al.*, 2013 ▸). The previously reported synthetic strategy employed a 25-fold excess of the *Ln*^III^ salt in order to promote the solubility of cucurbit[6]uril. In this work we introduced ultrasonication before placing the reaction mixture in hydro­thermal conditions, which allowed the excess of the *Ln*^III^ salt needed for the cucurbit[6]uril to be dissolved to be decreased.

The obtained complex [Pr(**CB6**)(NO_3_)(H_2_O)_5_](NO_3_)_2_·9.56 H_2_O (**1**) crystallizes in the *P*2_1_*/n* space group, while previously reported complexes obtained as outcomes of inter­actions between Ln(NO_3_)_3_ and cucurbit[6]uril crystallized in the ortho­rhom­bic *Pna*2_1_ space group (*Ln* = Gd, Dy, Ho and Yb) or in the monoclinic *P*2_1_/*n* space group in the case of *Ln*^III^ (Samsonenko *et al.*, 2002 ▸; Ren *et al.*, 2013 ▸). The different space group in the case of **1** may be caused by a variation of the synthetic conditions or the different lanthanide(III) radii.

**Figure 1:**
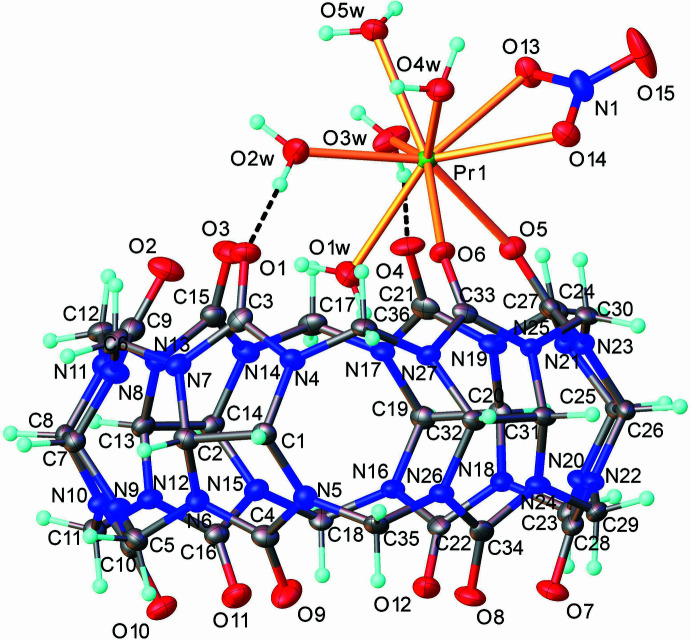
The mol­ecular structure of the [Pr(CB6)(NO_3_)(H_2_O)_5_]^2+^ cation (mol­ecule *A*) with selected atom-labeling scheme and displacement ellipsoids drawn at the 50% level.

The unit cell of complex **1** contains eight Pr^III^–cucurbituril cationic [Pr(**CB6**)(NO_3_)(H_2_O)_5_]^2+^ complex mol­ecules (Fig. 1 ▸) per unit cell, non-coordinated water mol­ecules and two nitrate anions per complex cation for charge balance. There are two crystallographically independent complex cations in the asymmetric unit of **1**, however, the differences in the coordination environments of the Pr^III^ ions are minor. The Pr^III^ ions in the complex **1** are nona­coordinated. Two coordination positions of the Pr^III^ ions are occupied by two carbonyl oxygen atoms from the coordinated **CB6** ligands, two positions contain oxygen atoms from the bidentate nitrate anions and the remaining five positions are occupied by oxygen atoms from the coordinated water mol­ecules. The macrocyclic cucurbit[6]uril coordinates in bidentate mode, which is typical for *Ln*^III^–cucurbit[*n*]uril complexes without additional ligands. The carbonyl oxygen atoms on the opposite side of the macrocycle remain uncoordinated.

**Figure 2:**
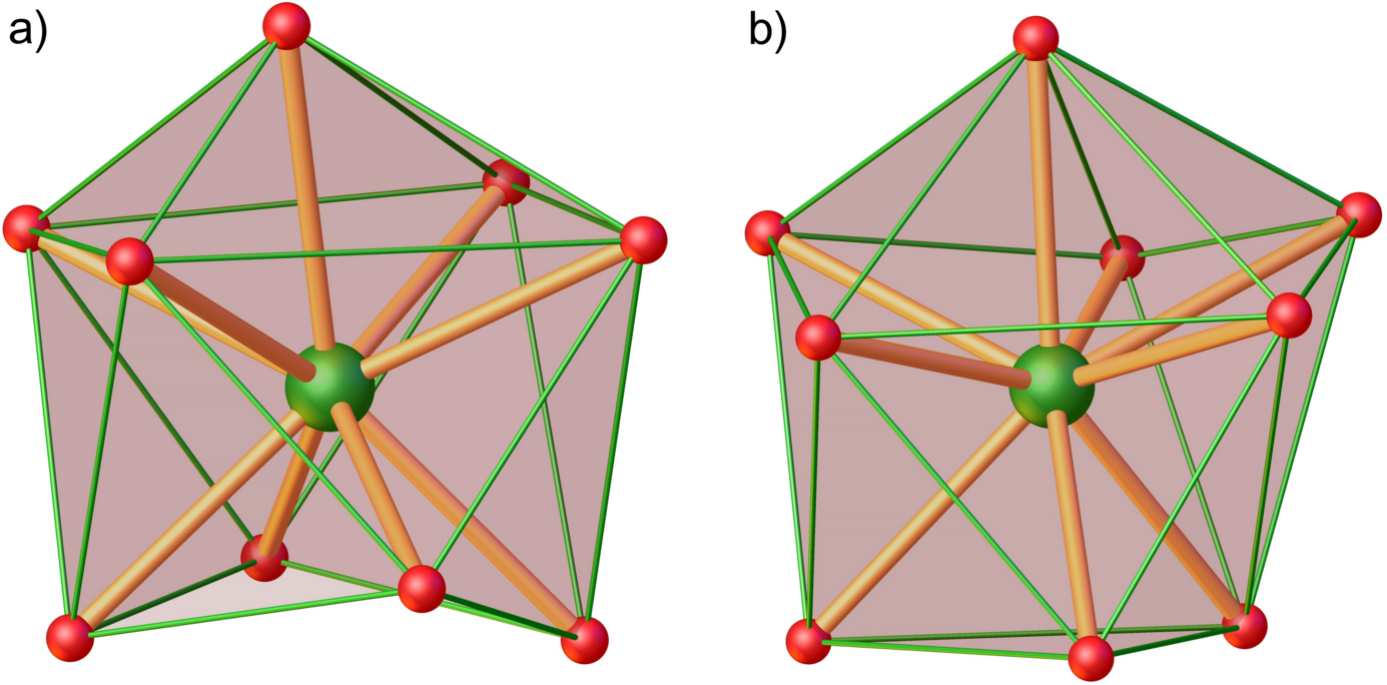
Coordination environments of the (*a*) Pr1*A* and (*b*) Pr1*B* ions in complex **1**.

The Pr—O_carbon­yl_ bond distances in complex **1** are typical for cucurbit[6]uril complexes with *Ln*^III^ ions (Samsonenko *et al.*, 2002 ▸; Ren *et al.*, 2013 ▸; da Silva *et al.*, 2014 ▸; Lin *et al.*, 2019 ▸). The observed bond distances between the Pr^III^ ions and the nitrate oxygen atoms are typical for bidentately coordinated nitrate anions to Pr^III^ ions (Pavlishchuk *et al.*, 2019 ▸). However, minor differences in the geometrical parameters of the coordination spheres of Pr1*A* and Pr1*B* are observed (Table 1 ▸, (Fig. 2 ▸). According to the calculations performed with *Shape 2.1* software (Casanova *et al.*, 2005 ▸, Table 2 ▸), the nona­coordinated Pr^III^ ions in complex **1** exhibit different geometries of the coordination environment: the Pr1*A* ions are located in a spherical capped square-anti­prismatic environment (CSAPR-9, *C*_4*v*_), while the geometry of the Pr1*B* ions is best described as a muffin polyhedron (MFF-9, *C_s_*).

## 3. Supra­molecular features

**Figure 3:**
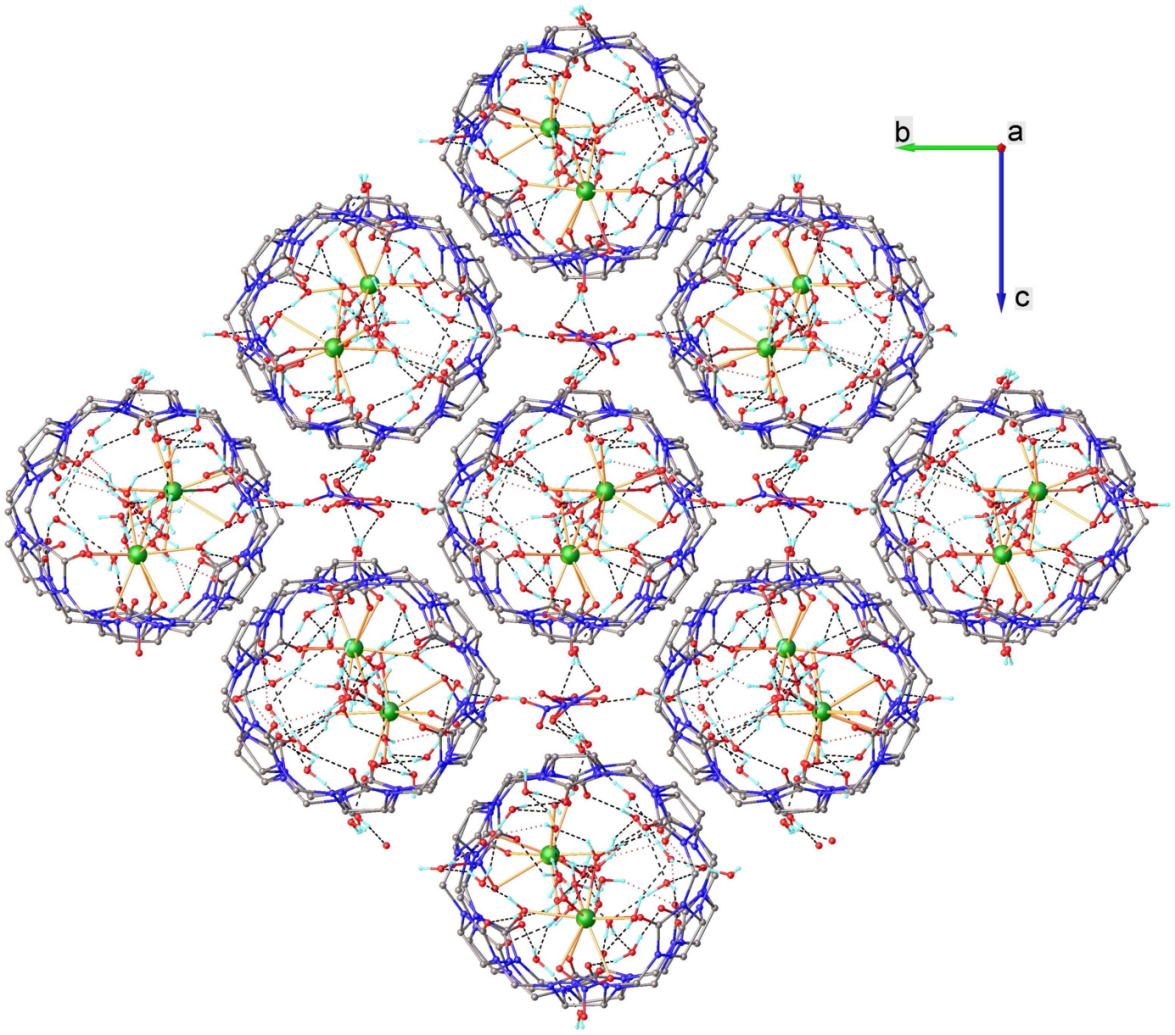
Fragment of the crystal structure viewed along the *a* axis.

The cationic fragments [Pr(CB6)(NO_3_)(H_2_O)_5_]^2+^ in complex **1** are linked to each other through an extended system of hydrogen bonds (Table 3 ▸, Figs. 3 ▸ and 4 ▸). The carbonyl oxygen atoms, which are located on opposite side of macrocycle with respect to the coordinated Pr^III^ ions are involved in the formation of an extended system of hydrogen-bonded water mol­ecules, which provide the supra­molecular organization of **1**. The carbonyl oxygen atoms O11*A*, O9*B* and O10*B* from the uncoordinated sides of the **CB6** ligands form hydrogen bonds with water mol­ecules in the coordination sphere of the Pr^III^ ions from adjacent complex cations (O11*A*–H4*WB*⋯O4*WB*, O11*A*—H5*WA*⋯O5*WB*, O9*B*—H5*WD*⋯O5*WA* and O10*B*—H4*WC*⋯·O4*WA*). In addition, there are intra­molecular hydrogen bonds that are formed between the carbonyl oxygen atoms located on the coordinated side of the **CB6** ligands with the water mol­ecules coordinated to the Pr^III^ ions from the same [Pr(**CB6**)(NO_3_)(H_2_O)_5_]^2+^ fragment (O1*A*—H2*WC*⋯O2*WA*, O4*A*—H3*WC*⋯O3*WA*, O1*B*—H2*WB*⋯O2*WB* and O4*B*—H3*WB*⋯O3*WB*). Other non-coordinated carbonyl oxygen atoms from **CB6** are involved in the formation of hydrogen bonds with non-coordinated water mol­ecules located between adjacent [Pr(**CB6**)(NO_3_)(H_2_O)_5_]^2+^ fragments (in complex cations with Pr1*A* ions: O2*A*—H17*E*⋯O17*W*, O3*A*—H17*W*⋯O17*W*, O3*A*—H15*A*⋯O15*W*, O7*A*—H4*F*⋯O4*W*, O8*A*—H3*WF*⋯O3*W*, O10*A*—H2*WE*⋯O2*W* and O12*A*—H11*F*⋯O11*W*; in complex cations with Pr1*B* ions: O2*B*—H12*F*⋯O12*W*, O3*B*—H12*E*⋯O12*W*, O7*B*—H19*C*⋯O19*W*, O8*B*—H16*A*⋯O16*W*, O11*B*—H14*C*⋯O14*W* and O12*B*—H24*F*⋯O24*W*). Water mol­ecules coordinated to Pr^III^ ions in **1** are also involved in the formation of hydrogen bonds with non-coordinated water mol­ecules (water mol­ecules coordinated to Pr1*A*: O1*WA*—H1*WA⋯*O9*W*, O2*WA—*H2*WD*⋯O19*W*, O3*WA*—H3*WD⋯*O16*W*, O4*WA—*H4*WD⋯*O14*W*, O5*WA*—H5*WC⋯*O19*W*; water mol­ecules coordinated to Pr1*B*: O2*WB*—H2*WA*⋯O11*W*, O3*WB—*H3*W⋯*O2*W*, O4*WB—*H4*WA⋯*O1*W*, O5*WB*—H5*WB⋯*O4*W*).

In summary, we have synthesized a new Pr^III^ complex with the macrocyclic cucurbit[6]uril ligand, which crystallizes in space group *P*2_1_/*n*, while previously reported complexes with other lanthanide ions *Ln*^III^ = La, Gd, Dy, Ho and Yb crystallized in *P*2_1_/*n* or *Pna*2_1_. The crystal structure of **1** contains [Pr(CB6)(NO_3_)(H_2_O)_5_]^2+^ complex cations, two non-coordin­ated nitrates per cation and non-coordinated water mol­ecules. Subtle differences in the bond distances and angles in the Pr^III^ coordination spheres leads to the observation of two crystallographically different types of Pr^III^ ions. The composition of the coordination sphere of two types of nona­coordinated Pr^III^ ions in **1** is the same, however the symmetry of the coordination environment of Pr1*A* and Pr1*B* ions is different.

## 4. Synthesis and crystallization

**Figure 5:**
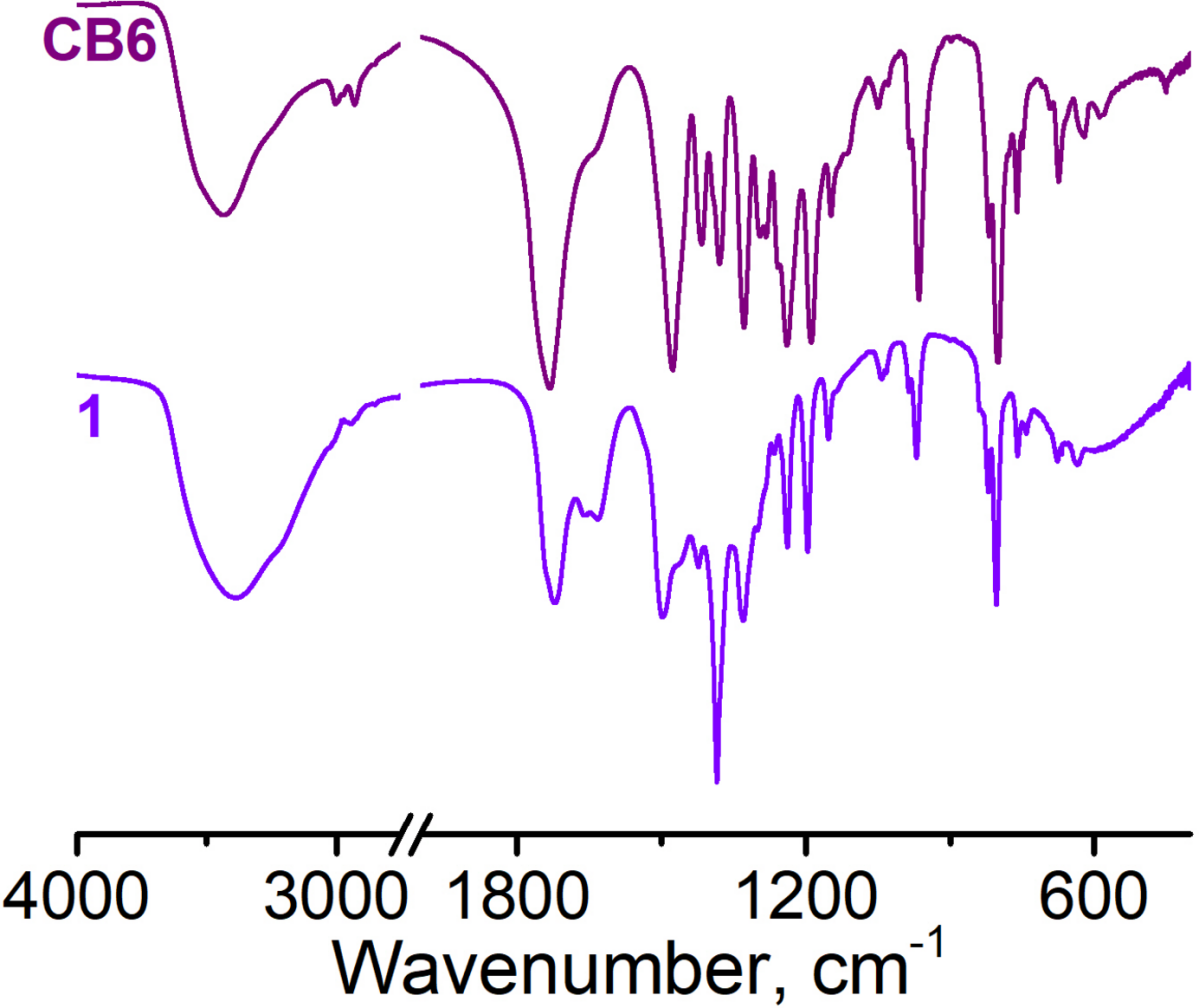
IR spectra of **CB6** and complex **1** recorded in a KBr pellet.

Cucurbit[6]uril was obtained by a modified procedure (Zbruyev *et al.*, 2023 ▸). Cucurbit[6]uril (C_36_H_36_N_24_O_12_·10H_2_O, **CB6**, 11.8 mg, 0.01 mmol) and Pr(NO_3_)_3_·6H_2_O (42 mg, 0.1 mmol) were placed in a closed vial containing 3 mL of water and ultrasonicated for the enhancement of macrocycle solubility. The obtained suspension was heated for 1 h at 358 K in a sand bath, which was accompanied by dissolution of macrocyclic ligand. Afterwards the observed clear solution had been heated at 363 K for 2 h. Slow cooling in the sand bath led to the formation of colorless crystals of **1** in one day. IR spectra of **CB6** and complex **1** are shown in Fig. 5 ▸.

## 5. Refinement

Crystal data, data collection and structure refinement details are summarized in Table 4 ▸. H atoms were placed in calculated positions and refined as riding.

## Supplementary Material

Crystal structure: contains datablock(s) I. DOI: 10.1107/S2056989024005760/ex2083sup1.cif

Structure factors: contains datablock(s) I. DOI: 10.1107/S2056989024005760/ex2083Isup4.hkl

CCDC reference: 2362791

Additional supporting information:  crystallographic information; 3D view; checkCIF report

## Figures and Tables

**Figure 4:**
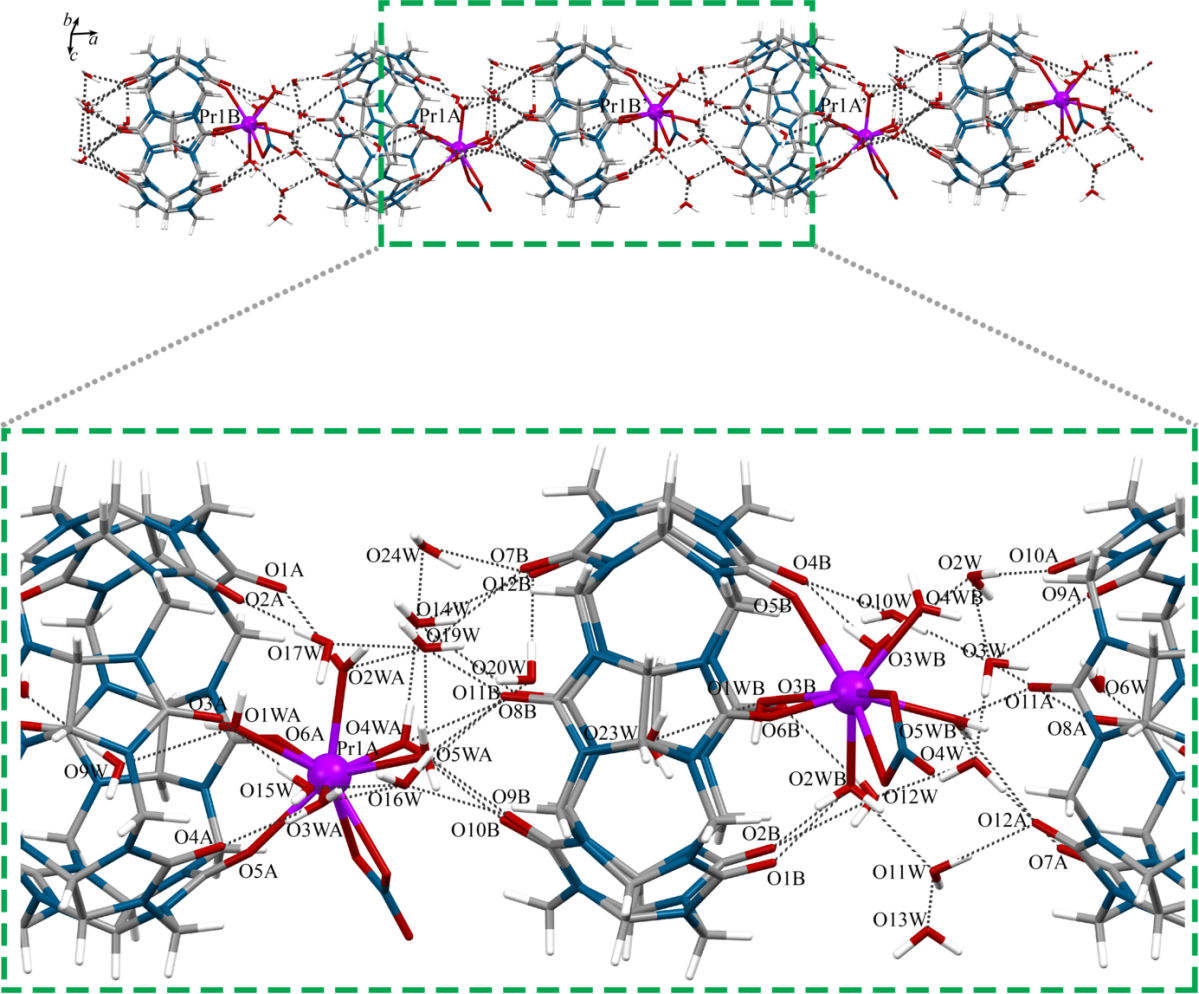
System of hydrogen bonds connecting the [Pr(CB6)(NO_3_)(H_2_O)_5_]^2+^ units in complex **1**.

**Table 1:** Selected geometric parameters (Å, °)

Pr1*A*—O1*WA*	2.544 (2)	Pr1*B*—O1*WB*	2.553 (3)
Pr1*A*—O2*WA*	2.449 (3)	Pr1*B*—O2*WB*	2.438 (2)
Pr1*A*—O3*WA*	2.442 (2)	Pr1*B*—O3*WB*	2.448 (2)
Pr1*B*—O4*WB*	2.527 (3)	Pr1*B*—O4*WB*	2.527 (3)
Pr1*A*—O5*A*	2.493 (2)	Pr1*B*—O5*B*	2.519 (2)
Pr1*A*—O5*WA*	2.483 (2)	Pr1*B*—O5*WB*	2.476 (2)
Pr1*A*—O6*A*	2.466 (2)	Pr1*B*—O6*B*	2.460 (2)
Pr1*A*—O13*A*	2.571 (2)	Pr1*B*—O13*B*	2.651 (3)
Pr1*A*—O14*A*	2.613 (2)	Pr1*B*—O14*B*	2.572 (2)
			
O2*WA*—Pr1*A*—O1*WA*	68.01 (9)	O2*WB*—Pr1*B*—O13*B*	68.61 (8)
O2*WA*—Pr1*A*—O4*WA*	77.25 (8)	O2*WB*—Pr1*B*—O3*WB*	84.55 (8)
O2*WA*—Pr1*A*—O5*WA*	67.92 (8)	O2*WB*—Pr1*B*—O6*B*	96.37 (8)
O2*WA*—Pr1*A*—O6*A*	84.64 (8)	O2*WB*—Pr1*B*—O1*WB*	67.77 (9)
O3*WA*—Pr1*A*—O1*WA*	73.57 (9)	O2*WB*—Pr1*B*—O5*WB*	71.46 (8)
O3*WA*—Pr1*A*—O2*WA*	93.13 (9)	O3*WB*—Pr1*B*—O5*WB*	71.21 (8)
O3*WA*—Pr1*A*—O5*WA*	70.71 (8)	O3*WB*—Pr1*B*—O5*B*	78.97 (8)
O3*WA*—Pr1*A*—O5*A*	77.35 (8)	O3*WB*—Pr1*B*—O4*WB*	76.07 (9)
O3*WA*—Pr1*A*—O13*A*	73.92 (8)	O3*WB*—Pr1*B*—O1*WB*	71.69 (9)
O4*WA*—Pr1*A*—O13*A*	88.91 (8)	O3*WB*—Pr1*B*—O1*WB*	71.69 (9)
O4*WA*—Pr1*A*—O14*A*	65.33 (8)	O4*WB*—Pr1*B*—O14*B*	65.36 (8)
O5*WA*—Pr1*A*—O4*WA*	71.24 (8)	O5*B*—Pr1*B*—O4*WB*	70.75 (8)
O5*WA*—Pr1*A*—O13*A*	70.72 (8)	O5*B*—Pr1*B*—O1*WB*	71.12 (9)
O5*A*—Pr1*A*—O1*WA*	72.17 (8)	O5*WB*—Pr1*B*—O4*WB*	69.88 (8)
O5*A*—Pr1*A*—O13*A*	76.26 (8)	O5*WB*—Pr1*B*—O14*B*	82.62 (8)
O5*A*—Pr1*A*—O14*A*	71.00 (8)	O5*WB*—Pr1*B*—O13*B*	71.53 (8)
O6*A*—Pr1*A*—O1*WA*	74.26 (8)	O6*B*—Pr1*B*—O1*WB*	75.85 (9)
O6*A*—Pr1*A*—O4*WA*	70.85 (8)	O6*B*—Pr1*B*—O5*B*	77.22 (8)
O6*A*—Pr1*A*—O5*A*	82.86 (8)	O6*B*—Pr1*B*—O14*B*	71.49 (8)
O6*A*—Pr1*A*—O14*A*	78.55 (7)	O6*B*—Pr1*B*—O13*B*	71.15 (8)
O13*A*—Pr1*A*—O14*A*	49.19 (7)	O14*B*—Pr1*B*—O13*B*	48.49 (8)

**Table 2:** Continuous shape calculations for nona­coordinated Pr^III^ ions in complex **1** performed with *Shape 2.1* software (Casanova *et al.*, 2005 ▸) Nonacoordinated ions: EP-9 – enneagon (*D*9*h*); OPY-9 – octa­gonal pyramid (*C*8*v*); HBPY-9 – hepta­gonal bipyramid (*D*7*h*); JTC-9 – Johnson triangular cupola J3 (*C*3*v*); JCCU-9 – capped cube J8 (*C*4*v*); CCU-9 – spherical-relaxed capped cube (*C*4*v*); JCSAPR-9 – capped square anti­prism J10 (C4*v*); CSAPR-9 – spherical capped square anti­prism (*C*4*v*); JTCTPR-9 – tricapped trigonal prism J51 (*D*3*h*); TCTPR-9 – spherical tricapped trigonal prism (*C*3*h*); JTDIC-9 – tridiminished icosa­hedron J63 (*C*3*v*); HH-9 – hula hoop (*C*2*v*); MFF-9 – muffin (*Cs*).

	Pr1*A*	Pr1*B*
EP-9	36.646	33.312
OPY-9	21.958	21.971
HBPY-9	18.179	15.434
JTC-9	16.494	14.581
JCCU-9	9.826	8.982
CCU-9	8.542	7.654
JCSAPR-9	2.258	3.376
CSAPR-9	**1.150**	2.289
JTCTPR-9	2.890	3.350
TCTPR-9	1.506	2.654
JTDIC-9	13.110	13.482
HH-9	10.200	8.067
MFF-9	1.198	**1.787**

**Table 3:** Hydrogen-bond geometry (Å, °)

*D*—H⋯*A*	*D*—H	H⋯*A*	*D*⋯*A*	*D*—H⋯*A*
O1*WB*—H1*WA*⋯O3*B*	0.90	2.50	3.378 (4)	169
O2*WB*—H2*WA*⋯O11*W*^i^	0.87	1.85	2.674 (3)	157
O2*WB*—H2*WB*⋯O1*B*	0.87	2.07	2.865 (3)	150
O2*WB*—H2*WB*⋯O2*B*	0.87	2.46	2.894 (4)	111
O3*WB*—H3*WA*⋯O2*W*^i^	0.87	1.88	2.709 (4)	158
O3*WB*—H3*WB*⋯O4*B*	0.87	1.89	2.710 (3)	156
O4*WB*—H4*WA*⋯O1*W*^i^	0.87	2.01	2.729 (4)	139
O4*WB*—H4*WB*⋯O10*A*^ii^	0.87	2.48	3.211 (3)	142
O4*WB*—H4*WB*⋯O11*A*^ii^	0.87	2.60	3.206 (3)	127
O5*WB*—H5*WA*⋯O11*A*^ii^	0.87	2.08	2.798 (4)	140
O5*WB*—H5*WA*⋯O12*A*^ii^	0.87	2.44	3.018 (3)	124
O5*WB*—H5*WB*⋯O4*W*^i^	0.87	1.86	2.712 (4)	167
O1*WA*—H1*WC*⋯O2*A*	0.86	2.41	3.251 (4)	167
O1*WA*—H1*WD*⋯O7*W*^c^	0.85	1.99	2.749 (17)	148
O1*WA*—H1*WD*⋯O8*W*^a^	0.85	2.25	2.751 (9)	118
O2*WA*—H2*WC*⋯O1*A*	0.87	1.80	2.667 (3)	179
O2*WA*—H2*WD*⋯O19*W*^iii^	0.87	1.95	2.723 (4)	147
O3*WA*—H3*WC*⋯O4*A*	0.87	1.87	2.723 (3)	166
O3*WA*—H3*WD*⋯O16*W*	0.87	1.93	2.733 (4)	153
O4*WA*—H4*WC*⋯O10*B*^iv^	0.87	2.05	2.905 (3)	167
O4*WA*—H4*WD*⋯O14*W*^iii^	0.85	1.93	2.743 (4)	159
O5*WA*—H5*WC*⋯O19*W*^iii^	0.87	1.93	2.766 (5)	159
O5*WA*—H5*WD*⋯O9*B*^iv^	0.87	1.97	2.676 (3)	137
O15*W*—H15*A*⋯O3*A*	0.87	1.97	2.837 (4)	174
O15*W*—H15*B*⋯O16*B*	0.87	2.19	3.019 (5)	159
O15*W*—H15*B*⋯O18*B*	0.87	2.39	3.128 (5)	143
O15*W*—H15*B*⋯N2*B*	0.87	2.53	3.394 (5)	173
O1*W*—H1*WE*⋯O21*B*	0.87	1.92	2.768 (4)	165
O1*W*—H1*WF*⋯O16*A*	0.87	1.89	2.750 (4)	170
O17*W*^b^—H17*E*^b^⋯O2*A*^v^	0.87	2.07	2.913 (6)	163
O17*W*^b^—H17*F*^b^⋯O3*A*^v^	0.87	2.46	3.045 (9)	126
O3*W*—H3*WE*⋯O4*W*	0.87	1.86	2.734 (4)	177
O3*W*—H3*WF*⋯O8*A*^vi^	0.87	1.98	2.764 (3)	150
O2*W*—H2*WE*⋯O9*A*^vi^	0.87	2.41	3.047 (3)	131
O2*W*—H2*WE*⋯O3*W*	0.87	2.06	2.850 (4)	151
O2*W*—H2*WF*⋯O10*A*^vi^	0.87	1.96	2.822 (3)	173
O21*W*^a^—H21*A*^a^⋯O10*B*	0.88	2.41	3.181 (9)	145
O21*W*^a^—H21*B*^a^⋯O11*B*	0.90	2.36	3.213 (9)	157
O21*W*^a^—H21*B*^a^⋯O12*B*	0.90	2.60	3.127 (10)	118
O19*W*—H19*C*⋯O7*B*^i^	0.87	2.29	2.856 (4)	123
O19*W*—H19*C*⋯O8*B*^i^	0.87	2.28	2.939 (4)	132
O19*W*—H19*D*⋯O17*W*^b^	0.87	2.16	2.813 (7)	132
O19*W*—H19*D*⋯O18*W*^a^	0.87	1.70	2.54 (2)	160
O14*W*—H14*C*⋯O11*B*^i^	0.87	2.42	2.870 (4)	112
O14*W*—H14*C*⋯O12*B*^i^	0.87	2.21	3.024 (4)	156
O14*W*—H14*D*⋯O24*W*	0.85	2.25	2.913 (5)	135
O6*W*—H6*WA*⋯O9*A*	0.86 (2)	2.29 (2)	3.101 (5)	159 (4)
O6*W*—H6*WB*⋯O10*A*	0.87 (2)	2.31 (2)	3.117 (5)	155 (4)
O16*W*—H16*A*⋯O8*B*^iv^	0.87	2.17	3.014 (4)	163
O16*W*—H16*B*⋯O15*W*	0.87	1.93	2.723 (4)	152
O7*W*^c^—H7*WA*^c^⋯O6*W*	0.89	1.79	2.673 (17)	170
O4*W*—H4*WE*⋯O12*W*	0.87	1.89	2.744 (4)	166
O4*W*—H4*WF*⋯O7*A*^vi^	0.87	1.91	2.777 (3)	177
O8*W*^a^—H8*WA*^a^⋯O6*W*	0.87	1.93	2.737 (9)	153
O8*W*^a^—H8*WB*^a^⋯N21*A*	0.87	2.66	3.316 (9)	133
O18*W*^a^—H18*E*^a^⋯O2*A*^v^	0.87	2.08	2.94 (2)	171
O18*W*^a^—H18*F*^a^⋯O25*W*	0.87	2.35	2.79 (4)	111
O9*W*^b^—H9*WA*^b^⋯N19*A*	0.83	2.69	3.411 (10)	146
O9*W*^b^—H9*WB*^b^⋯O6*W*	0.86	1.94	2.805 (10)	177
O5*W*—H5*WE*⋯O14*W*	0.90	1.58	2.440 (11)	158
O5*W*—H5*WF*⋯O20*B*^i^	0.87	1.99	2.786 (12)	151
O10*W*—H10*A*⋯O4*B*^i^	0.87	2.21	2.981 (3)	148
O10*W*—H10*B*⋯O3*W*	0.87	1.83	2.697 (4)	175
O20*W*^b^—H20*C*^b^⋯O12*B*	0.87	2.00	2.86 (2)	167
O20*W*^b^—H20*D*^b^⋯O5*WA*^vii^	0.87	2.38	3.00 (2)	128
O20*W*^b^—H20*D*^b^⋯O19*W*^i^	0.87	2.37	3.20 (4)	160
O11*W*—H11*E*⋯O13*W*	0.87	1.91	2.749 (4)	161
O11*W*—H11*F*⋯O12*A*^vi^	0.87	1.90	2.755 (4)	168
O22*W*^a^—H22*A*^a^⋯O21*W*^a^	0.87	1.80	2.670 (11)	179
O22*W*^a^—H22*B*^a^⋯O1*WB*	0.87	1.93	2.763 (8)	161
O12*W*—H12*E*⋯O3*B*^i^	0.87	2.05	2.913 (4)	169
O12*W*—H12*F*⋯O2*B*^i^	0.87	2.15	2.918 (4)	147
O25*W*—H25*C*⋯O21*A*	0.87	2.02	2.872 (5)	166
O25*W*—H25*D*⋯O18*B*^viii^	0.87	2.09	2.953 (6)	170
O13*W*—H13*C*⋯O17*B*	0.87	2.03	2.884 (5)	165
O13*W*—H13*D*⋯O21*A*^ix^	0.87	1.96	2.800 (4)	163
O24*W*—H24*E*⋯O25*W*	0.87	1.93	2.790 (5)	171
O24*W*—H24*F*⋯O12*B*^i^	0.87	2.17	2.839 (4)	133

Symmetry codes: (i) 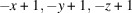; (ii) 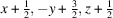; (iii) 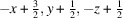; (iv) 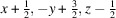; (v) 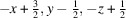; (vi) 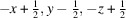; (vii) 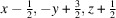; (viii) 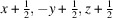; (ix) 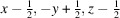.

**Table 4:** Experimental details

Crystal data	
Chemical formula	[Pr(NO_3_)(C_36_H_36_N_24_O_12_)(H_2_O)_5_](NO_3_)_2_·9.56H_2_O
*M*_r_	1586.05
Crystal system, space group	Monoclinic, *P*2_1_/*n*
Temperature (K)	180
*a*, *b*, *c* (Å)	24.1937 (3), 17.01202 (19), 28.6422 (3)
β (°)	90.5965 (11)
*V* (Å^3^)	11788.0 (2)
*Z*	8
Radiation type	Mo *K*α
μ (mm^−1^)	0.95
Crystal size (mm)	0.25 × 0.10 × 0.05

Data collection	
Diffractometer	Rigaku Oxford Diffraction Xcalibur, Eos
Absorption correction	Multi-scan (*CrysAlis PRO*; Rigaku OD, 2022 ▸)
*T*_min_, *T*_max_	0.892, 1.000
No. of measured, independent and observed [*I* > 2σ(*I*)] reflections	75330, 20820, 17014
*R*_int_	0.043
(sin θ/λ)_max_ (Å^−1^)	0.595

Refinement	
*R*[*F*^2^ > 2σ(*F*^2^)], *wR*(*F*^2^), *S*	0.037, 0.087, 1.04
No. of reflections	20820
No. of parameters	1843
No. of restraints	84
H-atom treatment	H atoms treated by a mixture of independent and constrained refinement
Δρ_max_, Δρ_min_ (e Å^−3^)	1.26, −0.64

Computer programs: *CrysAlis PRO* (Rigaku OD, 2022 ▸), *SHELXT* (Sheldrick, 2015*a* ▸), *SHELXL2019/2* (Sheldrick, 2015*b* ▸) and *OLEX2* (Dolomanov *et al.*, 2009 ▸).

## supplementary crystallographic information

### Pentaaqua(cucurbit[6]uril-κ2O,O')(nitrato-κ2O,O')praseodymium(III) dinitrate 9.56-hydrate Crystal data

**Table:**

[Pr(NO_3_)(C_36_H_36_N_24_O_12_)(H_2_O)_5_](NO_3_)_2_·9.56H_2_O	*F*(000) = 6508
*M**_r_* = 1586.05	*D*_x_ = 1.787 Mg m^−^^3^
Monoclinic, *P*2_1_/*n*	Mo *K*α radiation, λ = 0.71073 Å
*a* = 24.1937 (3) Å	Cell parameters from 28067 reflections
*b* = 17.01202 (19) Å	θ = 1.7–29.2°
*c* = 28.6422 (3) Å	µ = 0.95 mm^−^^1^
β = 90.5965 (11)°	*T* = 180 K
*V* = 11788.0 (2) Å^3^	Prism, clear light colourless
*Z* = 8	0.25 × 0.10 × 0.05 mm

### Pentaaqua(cucurbit[6]uril-κ2O,O')(nitrato-κ2O,O')praseodymium(III) dinitrate 9.56-hydrate Data collection

**Table:**

Rigaku Oxford Diffraction Xcalibur, Eos diffractometer	20820 independent reflections
Radiation source: fine-focus sealed X-ray tube, Enhance (Mo) X-ray Source	17014 reflections with *I* > 2σ(*I*)
Graphite monochromator	*R*_int_ = 0.043
Detector resolution: 16.1593 pixels mm^-1^	θ_max_ = 25.0°, θ_min_ = 1.7°
ω scans	*h* = −28→28
Absorption correction: multi-scan (CrysAlisPro; Rigaku OD, 2022)	*k* = −20→20
*T*_min_ = 0.892, *T*_max_ = 1.000	*l* = −34→34
75330 measured reflections	

### Pentaaqua(cucurbit[6]uril-κ2O,O')(nitrato-κ2O,O')praseodymium(III) dinitrate 9.56-hydrate Refinement

**Table:**

Refinement on *F*^2^	Primary atom site location: dual
Least-squares matrix: full	Hydrogen site location: mixed
*R*[*F*^2^ > 2σ(*F*^2^)] = 0.037	H atoms treated by a mixture of independent and constrained refinement
*wR*(*F*^2^) = 0.087	*w* = 1/[σ^2^(*F*_o_^2^) + (0.0337*P*)^2^ + 14.5091*P*] where *P* = (*F*_o_^2^ + 2*F*_c_^2^)/3
*S* = 1.04	(Δ/σ)_max_ = 0.003
20820 reflections	Δρ_max_ = 1.26 e Å^−^^3^
1843 parameters	Δρ_min_ = −0.64 e Å^−^^3^
84 restraints	

### Pentaaqua(cucurbit[6]uril-κ2O,O')(nitrato-κ2O,O')praseodymium(III) dinitrate 9.56-hydrate Special details

**Table:**

Geometry. All esds (except the esd in the dihedral angle between two l.s. planes) are estimated using the full covariance matrix. The cell esds are taken into account individually in the estimation of esds in distances, angles and torsion angles; correlations between esds in cell parameters are only used when they are defined by crystal symmetry. An approximate (isotropic) treatment of cell esds is used for estimating esds involving l.s. planes.

### Pentaaqua(cucurbit[6]uril-κ2O,O')(nitrato-κ2O,O')praseodymium(III) dinitrate 9.56-hydrate Fractional atomic coordinates and isotropic or equivalent isotropic displacement parameters (Å^2^)

**Table:**

	*x*	*y*	*z*	*U*_iso_*/*U*_eq_	Occ. (<1)
Pr1B	0.56080 (2)	0.81394 (2)	0.59862 (2)	0.01479 (5)	
O1WB	0.47590 (11)	0.73681 (17)	0.62441 (10)	0.0377 (7)	
H1WA	0.486182	0.690310	0.636326	0.057*	
H1WB	0.470192	0.751057	0.653367	0.057*	
O1B	0.48772 (10)	0.91183 (14)	0.71903 (10)	0.0270 (6)	
O2WB	0.55961 (10)	0.79322 (14)	0.68282 (9)	0.0227 (6)	
H2WA	0.586751	0.819146	0.695909	0.034*	
H2WB	0.529881	0.814445	0.694371	0.034*	
O2B	0.48542 (11)	0.72565 (15)	0.75053 (12)	0.0422 (8)	
O3WB	0.58270 (10)	0.67361 (13)	0.59506 (9)	0.0255 (6)	
H3WA	0.609936	0.666078	0.575809	0.038*	
H3WB	0.554996	0.648289	0.582458	0.038*	
O3B	0.49472 (10)	0.55689 (16)	0.67188 (9)	0.0328 (7)	
O4WB	0.62515 (10)	0.80282 (15)	0.53001 (9)	0.0282 (6)	
H4WA	0.616218	0.761685	0.513332	0.042*	
H4WB	0.658602	0.792674	0.540175	0.042*	
O4B	0.51371 (10)	0.55885 (13)	0.56426 (9)	0.0220 (6)	
O5WB	0.65703 (9)	0.79418 (14)	0.62651 (9)	0.0239 (6)	
H5WA	0.684114	0.827830	0.625512	0.036*	
H5WB	0.667755	0.759336	0.646922	0.036*	
O5B	0.50626 (9)	0.77305 (13)	0.52729 (8)	0.0178 (5)	
O6B	0.48547 (9)	0.91097 (14)	0.59600 (9)	0.0220 (6)	
O7B	0.25571 (10)	0.75673 (15)	0.50609 (10)	0.0313 (7)	
O8B	0.23920 (10)	0.93703 (15)	0.57119 (9)	0.0247 (6)	
O9B	0.24207 (10)	0.88557 (14)	0.68395 (9)	0.0265 (6)	
O10B	0.23623 (10)	0.69061 (14)	0.73399 (10)	0.0267 (6)	
O11B	0.24724 (10)	0.52504 (16)	0.66726 (9)	0.0299 (6)	
O12B	0.26210 (10)	0.56796 (15)	0.55534 (10)	0.0321 (7)	
O13B	0.59277 (11)	0.94176 (14)	0.64444 (9)	0.0287 (6)	
O14B	0.60014 (10)	0.94555 (14)	0.56987 (9)	0.0237 (6)	
O15B	0.63579 (10)	1.03954 (14)	0.61214 (9)	0.0272 (6)	
N1B	0.60973 (12)	0.97756 (17)	0.60905 (11)	0.0189 (7)	
N4B	0.41097 (11)	0.98499 (16)	0.69788 (10)	0.0163 (6)	
N5B	0.31183 (11)	0.97714 (16)	0.68493 (10)	0.0157 (6)	
N6B	0.30779 (11)	0.88978 (16)	0.74275 (10)	0.0171 (6)	
N7B	0.40668 (11)	0.90972 (16)	0.76095 (11)	0.0200 (7)	
N8B	0.40462 (11)	0.77192 (16)	0.78199 (11)	0.0189 (7)	
N9B	0.30465 (11)	0.75783 (15)	0.77427 (10)	0.0178 (7)	
N10B	0.31271 (11)	0.62898 (15)	0.76599 (10)	0.0162 (6)	
N11B	0.41256 (11)	0.64389 (15)	0.76862 (10)	0.0175 (6)	
N12B	0.32050 (11)	0.51116 (16)	0.71870 (10)	0.0176 (6)	
N13B	0.42078 (11)	0.52820 (16)	0.71929 (10)	0.0186 (7)	
N14B	0.42782 (12)	0.46547 (17)	0.65184 (10)	0.0194 (7)	
N15B	0.32735 (12)	0.45865 (17)	0.64833 (10)	0.0196 (7)	
N16B	0.33628 (12)	0.48466 (17)	0.56576 (10)	0.0201 (7)	
N17B	0.43743 (12)	0.47786 (16)	0.56773 (10)	0.0179 (6)	
N18B	0.33767 (12)	0.56903 (16)	0.50693 (11)	0.0199 (7)	
N19B	0.43849 (11)	0.57223 (16)	0.51452 (10)	0.0165 (6)	
N20B	0.33547 (12)	0.70226 (16)	0.47753 (11)	0.0196 (7)	
N21B	0.43552 (11)	0.70763 (15)	0.48855 (10)	0.0144 (6)	
N22B	0.33276 (11)	0.83151 (16)	0.48861 (10)	0.0170 (6)	
N23B	0.43332 (11)	0.83771 (15)	0.49156 (10)	0.0145 (6)	
N24B	0.32163 (11)	0.95249 (16)	0.53227 (10)	0.0190 (7)	
N25B	0.42278 (11)	0.95266 (16)	0.54014 (10)	0.0167 (6)	
N26B	0.31293 (11)	1.00684 (16)	0.60210 (10)	0.0164 (6)	
N27B	0.41203 (11)	0.99137 (15)	0.61323 (10)	0.0158 (6)	
C1B	0.35630 (13)	1.00089 (19)	0.71601 (12)	0.0140 (7)	
H1B	0.352366	1.056931	0.725973	0.017*	
C2B	0.35189 (13)	0.94338 (18)	0.75820 (12)	0.0149 (7)	
H2B	0.341992	0.971511	0.787609	0.018*	
C3B	0.43990 (14)	0.93228 (19)	0.72489 (13)	0.0186 (8)	
C4B	0.28340 (14)	0.91371 (19)	0.70194 (12)	0.0158 (8)	
C5B	0.27999 (14)	0.83490 (19)	0.77356 (13)	0.0188 (8)	
H5BA	0.280534	0.856499	0.805659	0.023*	
H5BB	0.240865	0.830080	0.763508	0.023*	
C6B	0.42404 (15)	0.84978 (19)	0.79379 (14)	0.0226 (8)	
H6BA	0.464928	0.849079	0.795425	0.027*	
H6BB	0.410342	0.863812	0.825156	0.027*	
C7B	0.35437 (14)	0.73954 (18)	0.80104 (13)	0.0168 (8)	
H7B	0.350236	0.752971	0.834817	0.020*	
C8B	0.36084 (13)	0.64994 (19)	0.79352 (12)	0.0156 (7)	
H8B	0.362203	0.620768	0.823821	0.019*	
C9B	0.43919 (15)	0.7150 (2)	0.76524 (14)	0.0227 (8)	
C10B	0.27990 (14)	0.69254 (19)	0.75570 (12)	0.0175 (8)	
C11B	0.29540 (14)	0.54941 (19)	0.75811 (12)	0.0175 (8)	
H11A	0.254788	0.548881	0.753514	0.021*	
H11B	0.303860	0.518325	0.786516	0.021*	
C12B	0.43993 (14)	0.57008 (19)	0.76091 (12)	0.0171 (8)	
H12A	0.434301	0.536036	0.788491	0.021*	
H12B	0.480117	0.579755	0.758213	0.021*	
C13B	0.37447 (14)	0.47439 (19)	0.71995 (12)	0.0166 (8)	
H13B	0.377848	0.435552	0.746033	0.020*	
C14B	0.37760 (14)	0.43374 (19)	0.67130 (12)	0.0160 (7)	
H14B	0.379409	0.375225	0.674280	0.019*	
C15B	0.45185 (14)	0.5212 (2)	0.67998 (13)	0.0199 (8)	
C16B	0.29387 (14)	0.50168 (19)	0.67675 (13)	0.0190 (8)	
C17B	0.45297 (14)	0.4360 (2)	0.60984 (12)	0.0178 (8)	
H17A	0.442744	0.379980	0.606026	0.021*	
H17B	0.493642	0.438575	0.613659	0.021*	
C18B	0.31179 (15)	0.4360 (2)	0.60153 (13)	0.0218 (8)	
H18A	0.271048	0.438609	0.598368	0.026*	
H18B	0.323017	0.380692	0.596402	0.026*	
C19B	0.38675 (14)	0.46397 (19)	0.54149 (12)	0.0168 (8)	
H19B	0.385156	0.409391	0.528591	0.020*	
C20B	0.38917 (14)	0.52679 (19)	0.50213 (12)	0.0171 (8)	
H20B	0.392401	0.502091	0.470596	0.021*	
C21B	0.46726 (14)	0.53940 (19)	0.55065 (12)	0.0159 (7)	
C22B	0.30784 (15)	0.5433 (2)	0.54383 (13)	0.0214 (8)	
C23B	0.31583 (15)	0.6223 (2)	0.47205 (13)	0.0221 (8)	
H23A	0.274979	0.622172	0.473686	0.026*	
H23B	0.326201	0.603010	0.440736	0.026*	
C24B	0.46224 (14)	0.63159 (18)	0.48440 (12)	0.0163 (7)	
H24A	0.459304	0.613640	0.451587	0.020*	
H24B	0.501984	0.637397	0.492208	0.020*	
C25B	0.38771 (13)	0.72842 (19)	0.45926 (12)	0.0156 (7)	
H25B	0.392731	0.711757	0.426097	0.019*	
C26B	0.38432 (13)	0.81872 (19)	0.46399 (12)	0.0156 (7)	
H26B	0.384405	0.845439	0.432896	0.019*	
C27B	0.46232 (14)	0.77303 (19)	0.50474 (12)	0.0153 (7)	
C28B	0.30308 (15)	0.7629 (2)	0.49235 (13)	0.0212 (8)	
C29B	0.30666 (14)	0.9083 (2)	0.49094 (12)	0.0193 (8)	
H29A	0.317092	0.938994	0.463023	0.023*	
H29B	0.266041	0.901314	0.490064	0.023*	
C30B	0.45060 (14)	0.91719 (19)	0.50051 (12)	0.0154 (7)	
H30A	0.443177	0.949365	0.472334	0.018*	
H30B	0.490978	0.917773	0.506459	0.018*	
C31B	0.37221 (14)	0.99832 (19)	0.53747 (12)	0.0177 (8)	
H31B	0.374513	1.041237	0.513708	0.021*	
C32B	0.36752 (14)	1.0314 (2)	0.58800 (12)	0.0170 (8)	
H32B	0.371819	1.089811	0.588916	0.020*	
C33B	0.44358 (14)	0.94841 (19)	0.58436 (12)	0.0164 (8)	
C34B	0.28648 (14)	0.96181 (19)	0.56926 (12)	0.0163 (8)	
C35B	0.29035 (14)	1.0259 (2)	0.64696 (12)	0.0184 (8)	
H35A	0.249667	1.019953	0.645373	0.022*	
H35B	0.298532	1.081663	0.654069	0.022*	
C36B	0.43400 (14)	1.0215 (2)	0.65738 (12)	0.0187 (8)	
H36A	0.474563	1.013763	0.658018	0.022*	
H36B	0.426873	1.078735	0.658897	0.022*	
Pr1A	0.57442 (2)	0.76863 (2)	0.18574 (2)	0.01388 (5)	
O1A	0.52959 (10)	0.90673 (14)	0.06042 (9)	0.0244 (6)	
O1WA	0.48655 (11)	0.74271 (16)	0.13871 (10)	0.0338 (7)	
H1WC	0.493989	0.736521	0.109566	0.051*	
H1WD	0.462049	0.777421	0.144625	0.051*	
O2A	0.49150 (11)	0.72148 (15)	0.02604 (10)	0.0319 (7)	
O2WA	0.58725 (11)	0.79270 (15)	0.10227 (9)	0.0295 (6)	
H2WC	0.568280	0.830052	0.088916	0.044*	
H2WD	0.607064	0.773022	0.079956	0.044*	
O3WA	0.56863 (10)	0.62637 (14)	0.17536 (10)	0.0307 (7)	
H3WC	0.538318	0.604139	0.184774	0.046*	
H3WD	0.584088	0.591259	0.157684	0.046*	
O3A	0.47393 (10)	0.53124 (15)	0.08121 (10)	0.0298 (6)	
O4A	0.47559 (10)	0.54094 (14)	0.19161 (10)	0.0258 (6)	
O4WA	0.64300 (10)	0.88067 (13)	0.18532 (9)	0.0232 (6)	
H4WC	0.674158	0.864967	0.197585	0.035*	
H4WD	0.648367	0.895498	0.157396	0.035*	
O5WA	0.66466 (9)	0.71843 (14)	0.15799 (9)	0.0224 (6)	
H5WC	0.660848	0.700271	0.129599	0.034*	
H5WD	0.674338	0.677470	0.174529	0.034*	
O5A	0.50058 (10)	0.73070 (13)	0.24148 (9)	0.0201 (5)	
O6A	0.52373 (9)	0.89438 (13)	0.18430 (8)	0.0197 (5)	
O7A	0.25431 (10)	0.80013 (15)	0.25809 (10)	0.0275 (6)	
O8A	0.27946 (10)	0.98195 (15)	0.19179 (9)	0.0270 (6)	
O9A	0.28105 (11)	0.95178 (16)	0.07980 (10)	0.0340 (7)	
O10A	0.24609 (10)	0.77112 (14)	0.01672 (9)	0.0246 (6)	
O11A	0.22893 (10)	0.60894 (15)	0.07673 (9)	0.0284 (6)	
O12A	0.23249 (10)	0.62070 (15)	0.19417 (9)	0.0262 (6)	
O13A	0.62804 (11)	0.70786 (14)	0.25481 (9)	0.0253 (6)	
O14A	0.59886 (10)	0.82552 (13)	0.26813 (9)	0.0217 (6)	
O15A	0.64832 (15)	0.76442 (17)	0.32079 (11)	0.0532 (10)	
N1A	0.62551 (13)	0.76602 (17)	0.28276 (11)	0.0250 (7)	
N4A	0.46216 (12)	0.99487 (16)	0.08181 (10)	0.0192 (7)	
N5A	0.36272 (11)	1.02021 (17)	0.08671 (10)	0.0199 (7)	
N6A	0.35073 (11)	0.93992 (16)	0.02598 (10)	0.0183 (6)	
N7A	0.45086 (12)	0.92786 (17)	0.01601 (11)	0.0204 (7)	
N8A	0.42725 (12)	0.79814 (16)	−0.01314 (11)	0.0220 (7)	
N9A	0.32820 (12)	0.81825 (16)	−0.01343 (10)	0.0186 (7)	
N10A	0.31330 (11)	0.68980 (16)	−0.01359 (10)	0.0183 (7)	
N11A	0.41303 (12)	0.66954 (16)	−0.00882 (11)	0.0202 (7)	
N12A	0.29677 (11)	0.56631 (16)	0.02689 (10)	0.0184 (7)	
N13A	0.39697 (12)	0.54957 (16)	0.03391 (10)	0.0186 (7)	
N14A	0.39014 (12)	0.47167 (17)	0.09562 (11)	0.0210 (7)	
N15A	0.29310 (12)	0.51386 (17)	0.09766 (10)	0.0199 (7)	
N16A	0.29088 (11)	0.51562 (16)	0.18255 (10)	0.0183 (7)	
N17A	0.38934 (11)	0.48661 (16)	0.17968 (10)	0.0183 (7)	
N18A	0.30528 (11)	0.59156 (17)	0.24436 (10)	0.0187 (7)	
N19A	0.40343 (11)	0.56116 (16)	0.24222 (11)	0.0192 (7)	
N20A	0.32296 (12)	0.71531 (17)	0.28327 (11)	0.0200 (7)	
N21A	0.42169 (11)	0.68743 (16)	0.27817 (10)	0.0174 (6)	
N22A	0.34140 (11)	0.84330 (16)	0.28023 (10)	0.0176 (6)	
N23A	0.43991 (11)	0.81477 (16)	0.27806 (10)	0.0157 (6)	
N24A	0.35820 (11)	0.96256 (16)	0.23646 (10)	0.0173 (6)	
N25A	0.45675 (11)	0.93532 (16)	0.23537 (10)	0.0154 (6)	
N26A	0.36452 (11)	1.03135 (16)	0.17075 (10)	0.0175 (6)	
N27A	0.46227 (11)	0.99413 (16)	0.16680 (10)	0.0171 (6)	
C1A	0.41290 (14)	1.0302 (2)	0.06082 (12)	0.0177 (8)	
H1A	0.419203	1.086604	0.052585	0.021*	
C2A	0.40301 (14)	0.97888 (19)	0.01633 (13)	0.0181 (8)	
H2A	0.400656	1.011773	−0.012555	0.022*	
C3A	0.48476 (15)	0.93862 (19)	0.05407 (13)	0.0188 (8)	
C4A	0.32705 (14)	0.9686 (2)	0.06553 (13)	0.0204 (8)	
C5A	0.31757 (14)	0.9012 (2)	−0.00957 (13)	0.0202 (8)	
H5AA	0.278013	0.909101	−0.002417	0.024*	
H5AB	0.324788	0.926356	−0.040077	0.024*	
C6A	0.46087 (15)	0.8674 (2)	−0.01824 (13)	0.0217 (8)	
H6AA	0.454236	0.889699	−0.049731	0.026*	
H6AB	0.500251	0.851832	−0.016240	0.026*	
C7A	0.37517 (14)	0.7875 (2)	−0.03791 (13)	0.0188 (8)	
H7A	0.376834	0.807493	−0.070695	0.023*	
C8A	0.36599 (14)	0.6974 (2)	−0.03618 (12)	0.0183 (8)	
H8A	0.365282	0.673857	−0.068160	0.022*	
C9A	0.44835 (15)	0.7287 (2)	0.00399 (13)	0.0220 (8)	
C10A	0.29131 (15)	0.7612 (2)	−0.00146 (12)	0.0187 (8)	
C11A	0.28239 (14)	0.6171 (2)	−0.01180 (12)	0.0191 (8)	
H11C	0.242538	0.629795	−0.010044	0.023*	
H11D	0.288281	0.587938	−0.041254	0.023*	
C12A	0.42587 (14)	0.5869 (2)	−0.00377 (13)	0.0204 (8)	
H12C	0.416412	0.559591	−0.033300	0.024*	
H12D	0.466140	0.581065	0.001627	0.024*	
C13A	0.34317 (14)	0.5129 (2)	0.02782 (13)	0.0184 (8)	
H13A	0.342463	0.476458	0.000443	0.022*	
C14A	0.33550 (14)	0.4682 (2)	0.07441 (12)	0.0183 (8)	
H14A	0.323378	0.412675	0.069001	0.022*	
C15A	0.42500 (15)	0.5194 (2)	0.07168 (13)	0.0208 (8)	
C16A	0.26900 (14)	0.5679 (2)	0.06818 (13)	0.0185 (8)	
C17A	0.40390 (15)	0.4378 (2)	0.14026 (13)	0.0213 (8)	
H17C	0.444165	0.427509	0.141487	0.026*	
H17D	0.384728	0.386668	0.143117	0.026*	
C18A	0.26556 (14)	0.4876 (2)	0.13986 (12)	0.0191 (8)	
H18C	0.226660	0.505640	0.138729	0.023*	
H18D	0.265280	0.429457	0.140346	0.023*	
C19A	0.33812 (14)	0.4794 (2)	0.20486 (12)	0.0190 (8)	
H19A	0.330427	0.423601	0.213605	0.023*	
C20A	0.34769 (14)	0.53184 (19)	0.24878 (12)	0.0170 (8)	
H20A	0.344245	0.501228	0.278418	0.020*	
C21A	0.42744 (15)	0.53089 (19)	0.20315 (13)	0.0196 (8)	
C22A	0.27237 (14)	0.5804 (2)	0.20566 (12)	0.0178 (8)	
C23A	0.29043 (14)	0.6442 (2)	0.28221 (13)	0.0218 (8)	
H23C	0.250884	0.658348	0.278920	0.026*	
H23D	0.295274	0.616259	0.312286	0.026*	
C24A	0.43424 (14)	0.60403 (19)	0.27711 (13)	0.0203 (8)	
H24C	0.474206	0.597253	0.271219	0.024*	
H24D	0.426414	0.581224	0.308146	0.024*	
C25A	0.37665 (13)	0.7209 (2)	0.30513 (12)	0.0165 (7)	
H25A	0.376373	0.699985	0.337761	0.020*	
C26A	0.38905 (13)	0.81014 (19)	0.30419 (12)	0.0165 (7)	
H26A	0.393595	0.832126	0.336384	0.020*	
C27A	0.45769 (14)	0.74376 (19)	0.26374 (12)	0.0157 (7)	
C28A	0.30109 (14)	0.7879 (2)	0.27206 (12)	0.0203 (8)	
C29A	0.33119 (14)	0.9268 (2)	0.27605 (12)	0.0191 (8)	
H29C	0.344162	0.953206	0.304968	0.023*	
H29D	0.290859	0.935683	0.273178	0.023*	
C30A	0.47198 (13)	0.88587 (19)	0.27464 (12)	0.0161 (7)	
H30C	0.511512	0.871647	0.272038	0.019*	
H30D	0.467635	0.916426	0.303790	0.019*	
C31A	0.41402 (13)	0.99428 (19)	0.23820 (12)	0.0166 (8)	
H31A	0.419762	1.030211	0.265448	0.020*	
C32A	0.41934 (13)	1.03807 (19)	0.19099 (12)	0.0158 (7)	
H32A	0.430147	1.094279	0.195670	0.019*	
C33A	0.48415 (13)	0.93666 (18)	0.19464 (12)	0.0155 (7)	
C34A	0.32906 (15)	0.9904 (2)	0.19885 (13)	0.0192 (8)	
C35A	0.34790 (14)	1.0668 (2)	0.12692 (12)	0.0193 (8)	
H35C	0.365426	1.119228	0.124362	0.023*	
H35D	0.307345	1.074600	0.126828	0.023*	
C36A	0.48871 (14)	1.0214 (2)	0.12462 (12)	0.0183 (8)	
H36C	0.527564	1.003145	0.124874	0.022*	
H36D	0.489178	1.079590	0.124805	0.022*	
O16B	0.54381 (15)	0.2575 (2)	0.11872 (14)	0.0720 (12)	
O17B	0.46355 (16)	0.20587 (19)	0.13009 (16)	0.0713 (12)	
O18B	0.47281 (16)	0.3073 (2)	0.08550 (15)	0.0740 (12)	
N2B	0.49486 (17)	0.2564 (2)	0.11405 (16)	0.0478 (10)	
O19B	0.21410 (11)	0.16709 (15)	0.62334 (10)	0.0316 (7)	
O20B	0.23535 (13)	0.28860 (16)	0.61011 (11)	0.0423 (8)	
O21B	0.29113 (11)	0.19422 (16)	0.58943 (10)	0.0322 (7)	
N3B	0.24697 (13)	0.21708 (19)	0.60798 (11)	0.0249 (7)	
O19A	0.70260 (12)	0.19623 (17)	0.61611 (11)	0.0423 (8)	
O20A	0.73974 (13)	0.31081 (16)	0.61681 (11)	0.0423 (8)	
O21A	0.79150 (12)	0.20722 (18)	0.61441 (12)	0.0455 (8)	
N3A	0.74391 (14)	0.23856 (18)	0.61561 (11)	0.0257 (7)	
O16A	0.45706 (13)	0.1619 (2)	0.57899 (13)	0.0570 (10)	
O17A	0.51151 (16)	0.2472 (2)	0.61215 (14)	0.0655 (10)	
O18A	0.53037 (12)	0.12449 (19)	0.61361 (10)	0.0424 (8)	
N2A	0.49932 (14)	0.1785 (2)	0.60257 (13)	0.0364 (9)	
O15W	0.55919 (11)	0.43362 (16)	0.11751 (10)	0.0359 (7)	
H15A	0.531808	0.463070	0.108221	0.054*	
H15B	0.545841	0.386081	0.117051	0.054*	
O1W	0.38077 (11)	0.27422 (14)	0.55348 (10)	0.0322 (7)	
H1WE	0.351200	0.256916	0.567085	0.048*	
H1WF	0.406991	0.243166	0.563275	0.048*	
O17W	0.9242 (2)	0.1000 (4)	0.4650 (3)	0.084 (3)	0.810 (13)
H17E	0.953798	0.128045	0.469856	0.125*	0.810 (13)
H17F	0.932326	0.070075	0.441486	0.125*	0.810 (13)
O3W	0.31358 (10)	0.45574 (16)	0.36281 (10)	0.0328 (7)	
H3WE	0.317282	0.412308	0.347052	0.049*	
H3WF	0.280852	0.472889	0.355052	0.049*	
O23W	0.3721 (7)	0.7829 (12)	0.6466 (11)	0.101 (6)	0.268 (13)
H23E	0.385269	0.798725	0.620035	0.151*	0.268 (13)
H23F	0.390731	0.804397	0.669369	0.151*	0.268 (13)
O2W	0.32994 (10)	0.38489 (14)	0.45206 (9)	0.0262 (6)	
H2WE	0.313484	0.409891	0.429402	0.039*	
H2WF	0.304996	0.352951	0.462630	0.039*	
O21W	0.2860 (5)	0.6953 (5)	0.6317 (4)	0.068 (3)	0.71 (2)
H21A	0.281586	0.712810	0.660446	0.102*	0.71 (2)
H21B	0.271467	0.646363	0.633036	0.102*	0.71 (2)
O19W	0.83588 (15)	0.1981 (2)	0.43782 (14)	0.0665 (11)	
H19C	0.803121	0.179963	0.444289	0.100*	
H19D	0.856591	0.182842	0.461129	0.100*	
O14W	0.84558 (13)	0.4649 (2)	0.39566 (12)	0.0575 (10)	
H14C	0.810849	0.454316	0.401063	0.086*	
H14D	0.863130	0.442404	0.417723	0.086*	
O6W	0.29069 (18)	0.7823 (3)	0.11899 (16)	0.0797 (13)	
H6WA	0.291 (3)	0.8322 (11)	0.1154 (17)	0.120*	
H6WB	0.276 (3)	0.765 (2)	0.0934 (10)	0.120*	
O16W	0.64638 (11)	0.52937 (15)	0.13768 (10)	0.0340 (7)	
H16A	0.668857	0.547816	0.116851	0.051*	
H16B	0.624094	0.498744	0.122059	0.051*	
O7W	0.3860 (7)	0.8012 (10)	0.1658 (6)	0.0459 (15)*	0.179 (3)
H7WA	0.355099	0.800048	0.148376	0.069*	0.179 (3)
H7WB	0.380089	0.780447	0.193037	0.069*	0.179 (3)
O4W	0.32609 (10)	0.32259 (15)	0.31050 (10)	0.0313 (7)	
H4WE	0.355673	0.332245	0.294487	0.047*	
H4WF	0.300503	0.314226	0.289477	0.047*	
O8W	0.3801 (3)	0.7352 (5)	0.1712 (3)	0.0459 (15)*	0.430 (3)
H8WA	0.346164	0.745262	0.162848	0.069*	0.430 (3)
H8WB	0.377194	0.700095	0.193128	0.069*	0.430 (3)
O18W	0.9063 (10)	0.1339 (17)	0.4920 (13)	0.085 (5)	0.190 (13)
H18E	0.936805	0.160753	0.490161	0.128*	0.190 (13)
H18F	0.898504	0.134003	0.521622	0.128*	0.190 (13)
O9W	0.3805 (4)	0.7046 (5)	0.1611 (3)	0.0459 (15)*	0.391 (3)
H9WA	0.371171	0.666792	0.177675	0.069*	0.391 (3)
H9WB	0.352882	0.729641	0.148865	0.069*	0.391 (3)
O5W	0.8489 (5)	0.6034 (6)	0.3737 (4)	0.043 (4)	0.272 (8)
H5WE	0.856544	0.554241	0.383207	0.065*	0.272 (8)
H5WF	0.817436	0.622181	0.383057	0.065*	0.272 (8)
O10W	0.40465 (11)	0.54046 (15)	0.38409 (10)	0.0323 (7)	
H10A	0.429569	0.504098	0.387889	0.048*	
H10B	0.374939	0.515039	0.375738	0.048*	
O20W	0.2621 (12)	0.7059 (13)	0.6121 (10)	0.076 (5)	0.29 (2)
H20C	0.261675	0.669053	0.591025	0.114*	0.29 (2)
H20D	0.233016	0.734203	0.605775	0.114*	0.29 (2)
O11W	0.36283 (10)	0.15384 (14)	0.25786 (9)	0.0272 (6)	
H11E	0.358043	0.190848	0.237224	0.041*	
H11F	0.330993	0.149829	0.271514	0.041*	
O22W	0.3694 (3)	0.7887 (5)	0.6070 (5)	0.103 (4)	0.570 (14)
H22A	0.342314	0.758387	0.615393	0.154*	0.570 (14)
H22B	0.399086	0.763617	0.615923	0.154*	0.570 (14)
O12W	0.41996 (11)	0.37574 (16)	0.26779 (11)	0.0382 (7)	
H12E	0.442509	0.394474	0.288857	0.057*	
H12F	0.437690	0.336314	0.255707	0.057*	
O25W	0.86965 (16)	0.2593 (3)	0.54626 (14)	0.0892 (14)	
H25C	0.842890	0.251026	0.565829	0.134*	
H25D	0.897800	0.234455	0.558301	0.134*	
O13W	0.36971 (13)	0.25594 (17)	0.18376 (12)	0.0483 (8)	
H13C	0.397488	0.249018	0.165131	0.072*	
H13D	0.341148	0.260256	0.165221	0.072*	
O24W	0.83946 (12)	0.37567 (19)	0.48225 (14)	0.0638 (11)	
H24E	0.847245	0.335749	0.500164	0.096*	
H24F	0.808034	0.363338	0.469114	0.096*	

### Pentaaqua(cucurbit[6]uril-κ2O,O')(nitrato-κ2O,O')praseodymium(III) dinitrate 9.56-hydrate Atomic displacement parameters (Å^2^)

**Table:**

	*U*^11^	*U*^22^	*U*^33^	*U*^12^	*U*^13^	*U*^23^
Pr1B	0.01164 (10)	0.01606 (10)	0.01665 (10)	−0.00069 (8)	−0.00064 (8)	0.00139 (8)
O1WB	0.0286 (16)	0.0493 (18)	0.0353 (17)	−0.0073 (14)	0.0040 (14)	0.0136 (14)
O1B	0.0118 (13)	0.0263 (14)	0.0427 (17)	0.0032 (11)	−0.0012 (13)	0.0008 (12)
O2WB	0.0164 (13)	0.0287 (14)	0.0228 (14)	0.0020 (11)	0.0017 (11)	0.0030 (11)
O2B	0.0200 (15)	0.0239 (15)	0.083 (3)	0.0007 (12)	0.0232 (16)	0.0093 (15)
O3WB	0.0148 (13)	0.0230 (14)	0.0385 (17)	−0.0014 (11)	−0.0002 (12)	−0.0035 (12)
O3B	0.0206 (14)	0.0501 (18)	0.0277 (16)	−0.0156 (13)	0.0074 (13)	−0.0098 (13)
O4WB	0.0199 (14)	0.0379 (16)	0.0268 (15)	−0.0019 (12)	−0.0010 (12)	−0.0058 (12)
O4B	0.0163 (13)	0.0201 (13)	0.0294 (15)	−0.0034 (10)	−0.0044 (12)	0.0006 (11)
O5WB	0.0123 (12)	0.0283 (14)	0.0311 (15)	−0.0053 (11)	−0.0022 (12)	0.0038 (11)
O5B	0.0134 (12)	0.0199 (13)	0.0199 (13)	0.0009 (10)	−0.0052 (11)	−0.0005 (10)
O6B	0.0143 (13)	0.0276 (14)	0.0239 (14)	0.0061 (11)	−0.0037 (11)	−0.0045 (11)
O7B	0.0166 (14)	0.0347 (16)	0.0427 (18)	−0.0043 (12)	0.0069 (13)	−0.0037 (13)
O8B	0.0129 (13)	0.0356 (15)	0.0258 (15)	−0.0029 (11)	0.0013 (12)	−0.0037 (12)
O9B	0.0214 (14)	0.0233 (14)	0.0345 (16)	−0.0075 (11)	−0.0105 (13)	0.0025 (12)
O10B	0.0186 (14)	0.0201 (14)	0.0410 (17)	−0.0008 (11)	−0.0116 (13)	−0.0008 (12)
O11B	0.0175 (14)	0.0448 (17)	0.0273 (16)	0.0083 (12)	−0.0027 (12)	−0.0083 (13)
O12B	0.0174 (14)	0.0368 (16)	0.0421 (18)	0.0071 (12)	0.0066 (13)	0.0077 (13)
O13B	0.0385 (16)	0.0286 (15)	0.0188 (14)	−0.0108 (12)	−0.0025 (13)	0.0069 (11)
O14B	0.0300 (15)	0.0231 (14)	0.0179 (14)	−0.0025 (11)	0.0035 (12)	0.0001 (11)
O15B	0.0271 (15)	0.0164 (13)	0.0381 (17)	−0.0051 (12)	−0.0029 (13)	0.0030 (11)
N1B	0.0132 (15)	0.0190 (16)	0.0245 (18)	0.0037 (13)	−0.0027 (14)	0.0035 (13)
N4B	0.0096 (14)	0.0213 (16)	0.0181 (16)	0.0003 (12)	−0.0002 (13)	−0.0004 (12)
N5B	0.0106 (14)	0.0173 (15)	0.0192 (16)	0.0003 (12)	−0.0025 (13)	0.0015 (12)
N6B	0.0135 (15)	0.0148 (15)	0.0231 (17)	−0.0038 (12)	−0.0030 (13)	0.0044 (12)
N7B	0.0120 (15)	0.0162 (15)	0.0317 (18)	0.0019 (12)	−0.0027 (14)	0.0050 (13)
N8B	0.0119 (15)	0.0145 (15)	0.0302 (18)	−0.0006 (12)	−0.0008 (14)	0.0016 (13)
N9B	0.0145 (15)	0.0129 (15)	0.0259 (17)	0.0009 (12)	−0.0036 (14)	0.0005 (12)
N10B	0.0153 (15)	0.0126 (15)	0.0206 (16)	−0.0009 (12)	−0.0030 (13)	−0.0015 (12)
N11B	0.0144 (15)	0.0130 (15)	0.0251 (17)	0.0026 (12)	0.0042 (13)	0.0029 (12)
N12B	0.0152 (15)	0.0190 (15)	0.0187 (16)	0.0029 (12)	−0.0002 (13)	−0.0029 (12)
N13B	0.0142 (15)	0.0232 (16)	0.0184 (16)	−0.0010 (12)	0.0019 (13)	−0.0017 (13)
N14B	0.0144 (15)	0.0228 (16)	0.0210 (17)	−0.0023 (13)	0.0029 (13)	−0.0018 (13)
N15B	0.0153 (15)	0.0247 (16)	0.0188 (16)	0.0020 (13)	−0.0011 (13)	−0.0032 (13)
N16B	0.0160 (15)	0.0236 (16)	0.0206 (17)	0.0022 (13)	0.0030 (14)	0.0040 (13)
N17B	0.0163 (15)	0.0181 (15)	0.0192 (16)	−0.0033 (12)	−0.0020 (13)	0.0019 (12)
N18B	0.0157 (15)	0.0197 (16)	0.0241 (17)	−0.0006 (13)	−0.0009 (14)	0.0049 (13)
N19B	0.0126 (15)	0.0153 (15)	0.0216 (16)	−0.0036 (12)	−0.0008 (13)	0.0015 (12)
N20B	0.0149 (15)	0.0190 (16)	0.0250 (17)	−0.0010 (12)	−0.0009 (14)	0.0015 (13)
N21B	0.0122 (14)	0.0141 (14)	0.0168 (15)	0.0009 (11)	−0.0030 (13)	−0.0014 (12)
N22B	0.0118 (14)	0.0191 (16)	0.0202 (16)	−0.0001 (12)	0.0019 (13)	−0.0015 (12)
N23B	0.0132 (14)	0.0135 (14)	0.0169 (15)	0.0015 (12)	−0.0022 (13)	0.0002 (12)
N24B	0.0123 (15)	0.0228 (16)	0.0217 (17)	−0.0004 (12)	−0.0009 (13)	−0.0062 (13)
N25B	0.0125 (15)	0.0189 (15)	0.0186 (16)	0.0045 (12)	0.0032 (13)	−0.0040 (12)
N26B	0.0135 (15)	0.0190 (15)	0.0166 (16)	−0.0003 (12)	0.0020 (13)	−0.0020 (12)
N27B	0.0119 (14)	0.0161 (15)	0.0193 (16)	0.0017 (12)	−0.0012 (13)	−0.0047 (12)
C1B	0.0125 (17)	0.0111 (17)	0.0186 (19)	−0.0010 (13)	0.0022 (15)	−0.0029 (14)
C2B	0.0134 (17)	0.0112 (17)	0.0200 (19)	−0.0005 (14)	−0.0016 (15)	−0.0018 (14)
C3B	0.0159 (19)	0.0117 (17)	0.028 (2)	−0.0026 (14)	−0.0030 (17)	−0.0062 (15)
C4B	0.0116 (17)	0.0124 (17)	0.024 (2)	0.0023 (14)	0.0023 (16)	−0.0033 (14)
C5B	0.0145 (18)	0.0152 (18)	0.027 (2)	0.0004 (14)	0.0018 (16)	0.0028 (15)
C6B	0.0175 (19)	0.0171 (19)	0.033 (2)	−0.0042 (15)	−0.0089 (17)	0.0037 (16)
C7B	0.0160 (18)	0.0143 (17)	0.0202 (19)	0.0004 (14)	−0.0007 (16)	0.0011 (14)
C8B	0.0138 (17)	0.0163 (18)	0.0166 (18)	0.0032 (14)	0.0005 (15)	0.0028 (14)
C9B	0.018 (2)	0.020 (2)	0.030 (2)	0.0020 (16)	−0.0002 (18)	0.0065 (16)
C10B	0.0156 (19)	0.0189 (19)	0.0182 (19)	0.0009 (15)	0.0021 (16)	0.0002 (15)
C11B	0.0162 (18)	0.0164 (18)	0.0200 (19)	−0.0032 (14)	0.0051 (16)	0.0015 (14)
C12B	0.0141 (18)	0.0176 (18)	0.0196 (19)	0.0050 (14)	−0.0009 (16)	0.0002 (14)
C13B	0.0178 (18)	0.0146 (18)	0.0174 (19)	0.0034 (14)	−0.0007 (16)	0.0037 (14)
C14B	0.0175 (18)	0.0132 (17)	0.0175 (19)	0.0001 (14)	0.0037 (16)	0.0047 (14)
C15B	0.0145 (19)	0.024 (2)	0.021 (2)	0.0042 (16)	−0.0012 (16)	0.0026 (16)
C16B	0.0164 (19)	0.0141 (18)	0.027 (2)	−0.0030 (15)	0.0010 (17)	0.0021 (15)
C17B	0.0147 (18)	0.0171 (18)	0.022 (2)	0.0049 (14)	0.0025 (16)	−0.0009 (15)
C18B	0.0176 (19)	0.023 (2)	0.025 (2)	−0.0042 (15)	0.0016 (17)	0.0016 (16)
C19B	0.0184 (18)	0.0147 (18)	0.0173 (19)	−0.0030 (14)	−0.0010 (16)	−0.0019 (14)
C20B	0.0168 (18)	0.0152 (18)	0.0194 (19)	−0.0037 (14)	0.0020 (16)	−0.0024 (14)
C21B	0.0148 (18)	0.0141 (17)	0.0187 (19)	0.0012 (14)	0.0025 (16)	−0.0059 (14)
C22B	0.018 (2)	0.022 (2)	0.024 (2)	−0.0058 (16)	−0.0028 (17)	−0.0006 (16)
C23B	0.0208 (19)	0.0208 (19)	0.024 (2)	−0.0050 (15)	−0.0067 (17)	0.0005 (16)
C24B	0.0146 (17)	0.0136 (17)	0.0206 (19)	0.0020 (14)	0.0005 (15)	−0.0020 (14)
C25B	0.0147 (17)	0.0180 (18)	0.0139 (18)	−0.0030 (14)	−0.0028 (15)	0.0019 (14)
C26B	0.0131 (17)	0.0193 (18)	0.0145 (18)	0.0019 (14)	−0.0014 (15)	−0.0009 (14)
C27B	0.0155 (18)	0.0188 (19)	0.0117 (17)	−0.0028 (15)	0.0050 (15)	0.0003 (14)
C28B	0.0157 (19)	0.029 (2)	0.0185 (19)	−0.0003 (16)	−0.0027 (16)	−0.0002 (16)
C29B	0.0122 (17)	0.026 (2)	0.019 (2)	0.0048 (15)	−0.0043 (16)	−0.0017 (15)
C30B	0.0141 (17)	0.0156 (18)	0.0165 (18)	−0.0005 (14)	0.0026 (15)	0.0017 (14)
C31B	0.0163 (18)	0.0168 (18)	0.0202 (19)	0.0039 (14)	0.0039 (16)	0.0013 (14)
C32B	0.0144 (18)	0.0160 (18)	0.0208 (19)	−0.0002 (14)	0.0038 (16)	0.0015 (14)
C33B	0.0131 (18)	0.0135 (17)	0.023 (2)	−0.0053 (14)	0.0002 (16)	−0.0022 (14)
C34B	0.0153 (18)	0.0158 (18)	0.0178 (19)	0.0075 (15)	0.0000 (16)	0.0021 (14)
C35B	0.0151 (18)	0.0198 (19)	0.0202 (19)	0.0044 (15)	0.0008 (16)	−0.0002 (15)
C36B	0.0135 (18)	0.0206 (19)	0.022 (2)	−0.0061 (15)	0.0016 (16)	−0.0049 (15)
Pr1A	0.01196 (10)	0.01339 (10)	0.01629 (10)	0.00072 (7)	−0.00024 (8)	−0.00015 (7)
O1A	0.0175 (13)	0.0225 (14)	0.0331 (16)	0.0061 (11)	−0.0009 (12)	0.0010 (11)
O1WA	0.0220 (14)	0.0508 (18)	0.0284 (16)	0.0002 (13)	−0.0039 (13)	−0.0070 (13)
O2A	0.0202 (14)	0.0316 (16)	0.0435 (18)	0.0002 (12)	−0.0116 (14)	−0.0023 (13)
O2WA	0.0275 (15)	0.0387 (16)	0.0222 (15)	0.0133 (12)	−0.0001 (12)	0.0067 (12)
O3WA	0.0223 (14)	0.0180 (14)	0.0519 (19)	−0.0063 (11)	0.0111 (14)	−0.0136 (12)
O3A	0.0158 (14)	0.0411 (16)	0.0324 (16)	−0.0032 (12)	−0.0023 (13)	0.0094 (13)
O4A	0.0115 (13)	0.0256 (14)	0.0404 (17)	−0.0005 (11)	0.0043 (12)	0.0014 (12)
O4WA	0.0187 (13)	0.0242 (14)	0.0265 (15)	−0.0014 (11)	0.0000 (12)	0.0035 (11)
O5WA	0.0190 (13)	0.0229 (13)	0.0252 (14)	0.0061 (11)	−0.0026 (12)	0.0020 (11)
O5A	0.0176 (13)	0.0185 (13)	0.0241 (14)	−0.0017 (10)	0.0017 (12)	−0.0010 (10)
O6A	0.0172 (13)	0.0203 (13)	0.0217 (14)	0.0065 (11)	0.0017 (11)	−0.0002 (10)
O7A	0.0135 (13)	0.0344 (15)	0.0343 (16)	−0.0016 (11)	−0.0076 (12)	0.0019 (12)
O8A	0.0121 (13)	0.0373 (16)	0.0315 (16)	−0.0004 (11)	−0.0043 (12)	0.0045 (12)
O9A	0.0208 (15)	0.0464 (18)	0.0349 (17)	−0.0139 (13)	0.0083 (13)	−0.0118 (13)
O10A	0.0187 (14)	0.0229 (14)	0.0322 (16)	0.0000 (11)	0.0071 (12)	−0.0056 (11)
O11A	0.0240 (14)	0.0324 (15)	0.0290 (16)	0.0104 (12)	0.0042 (13)	0.0016 (12)
O12A	0.0192 (14)	0.0340 (15)	0.0253 (15)	0.0094 (12)	−0.0025 (12)	−0.0016 (12)
O13A	0.0350 (16)	0.0160 (13)	0.0246 (15)	0.0037 (11)	−0.0073 (13)	0.0001 (11)
O14A	0.0235 (14)	0.0151 (13)	0.0265 (15)	0.0006 (11)	−0.0045 (12)	−0.0002 (11)
O15A	0.091 (3)	0.0393 (18)	0.0288 (18)	0.0010 (17)	−0.0338 (19)	0.0013 (14)
N1A	0.0325 (19)	0.0226 (18)	0.0197 (18)	−0.0064 (15)	−0.0063 (15)	0.0017 (14)
N4A	0.0167 (15)	0.0188 (16)	0.0221 (17)	0.0027 (12)	−0.0010 (14)	0.0006 (13)
N5A	0.0132 (15)	0.0244 (17)	0.0222 (17)	−0.0024 (13)	0.0001 (14)	−0.0028 (13)
N6A	0.0162 (15)	0.0192 (16)	0.0194 (16)	−0.0036 (12)	0.0015 (13)	−0.0014 (12)
N7A	0.0168 (16)	0.0209 (16)	0.0235 (17)	−0.0005 (13)	0.0025 (14)	−0.0027 (13)
N8A	0.0162 (16)	0.0192 (16)	0.0304 (18)	−0.0032 (13)	−0.0024 (14)	0.0000 (13)
N9A	0.0161 (15)	0.0156 (15)	0.0240 (17)	0.0006 (12)	0.0004 (14)	−0.0026 (12)
N10A	0.0135 (15)	0.0164 (15)	0.0251 (17)	−0.0013 (12)	0.0018 (13)	−0.0038 (13)
N11A	0.0141 (15)	0.0214 (16)	0.0250 (17)	−0.0003 (13)	−0.0025 (14)	0.0015 (13)
N12A	0.0131 (15)	0.0204 (16)	0.0219 (17)	−0.0003 (12)	0.0022 (13)	−0.0003 (13)
N13A	0.0151 (15)	0.0211 (16)	0.0195 (16)	−0.0009 (12)	0.0003 (13)	0.0010 (13)
N14A	0.0161 (16)	0.0227 (16)	0.0242 (17)	−0.0016 (13)	−0.0017 (14)	0.0043 (13)
N15A	0.0161 (16)	0.0238 (16)	0.0199 (17)	0.0020 (13)	0.0025 (14)	0.0006 (13)
N16A	0.0115 (15)	0.0227 (16)	0.0206 (16)	0.0010 (12)	−0.0007 (13)	−0.0038 (13)
N17A	0.0115 (15)	0.0236 (16)	0.0197 (16)	−0.0009 (12)	−0.0001 (13)	0.0021 (13)
N18A	0.0133 (15)	0.0248 (16)	0.0179 (16)	0.0001 (12)	0.0001 (13)	−0.0010 (13)
N19A	0.0125 (15)	0.0178 (15)	0.0272 (18)	−0.0029 (12)	−0.0025 (14)	−0.0009 (13)
N20A	0.0143 (15)	0.0221 (16)	0.0236 (17)	−0.0022 (13)	−0.0034 (14)	0.0004 (13)
N21A	0.0139 (15)	0.0160 (15)	0.0221 (16)	−0.0012 (12)	0.0008 (13)	−0.0012 (12)
N22A	0.0113 (14)	0.0229 (16)	0.0188 (16)	−0.0016 (12)	−0.0017 (13)	0.0018 (13)
N23A	0.0124 (14)	0.0170 (15)	0.0176 (16)	−0.0025 (12)	0.0024 (13)	−0.0017 (12)
N24A	0.0104 (14)	0.0200 (16)	0.0214 (17)	−0.0005 (12)	−0.0004 (13)	−0.0001 (12)
N25A	0.0125 (14)	0.0180 (15)	0.0156 (15)	0.0037 (12)	−0.0001 (13)	−0.0004 (12)
N26A	0.0121 (15)	0.0196 (16)	0.0207 (16)	−0.0001 (12)	−0.0005 (13)	0.0008 (12)
N27A	0.0146 (15)	0.0178 (15)	0.0190 (16)	0.0039 (12)	0.0016 (13)	0.0024 (12)
C1A	0.0189 (19)	0.0153 (18)	0.0188 (19)	0.0025 (14)	−0.0009 (16)	0.0044 (14)
C2A	0.0166 (18)	0.0164 (18)	0.021 (2)	−0.0005 (14)	−0.0016 (16)	0.0018 (15)
C3A	0.0202 (19)	0.0155 (18)	0.021 (2)	−0.0027 (15)	0.0060 (17)	0.0036 (15)
C4A	0.0168 (19)	0.0175 (19)	0.027 (2)	−0.0028 (15)	−0.0015 (17)	0.0002 (15)
C5A	0.0176 (19)	0.023 (2)	0.019 (2)	0.0007 (15)	−0.0028 (16)	0.0012 (15)
C6A	0.0188 (19)	0.021 (2)	0.025 (2)	−0.0049 (15)	0.0100 (17)	−0.0041 (16)
C7A	0.0173 (18)	0.0217 (19)	0.0175 (19)	−0.0014 (15)	0.0010 (16)	−0.0002 (15)
C8A	0.0185 (19)	0.0222 (19)	0.0140 (18)	0.0005 (15)	0.0004 (16)	−0.0046 (15)
C9A	0.019 (2)	0.025 (2)	0.022 (2)	−0.0030 (16)	0.0019 (17)	−0.0025 (16)
C10A	0.0175 (19)	0.023 (2)	0.0151 (18)	−0.0015 (15)	−0.0027 (16)	−0.0021 (15)
C11A	0.0168 (18)	0.0216 (19)	0.0188 (19)	−0.0023 (15)	−0.0010 (16)	−0.0032 (15)
C12A	0.0145 (18)	0.0210 (19)	0.026 (2)	0.0019 (15)	0.0025 (16)	−0.0003 (15)
C13A	0.0163 (18)	0.0184 (18)	0.021 (2)	0.0013 (15)	0.0022 (16)	−0.0038 (15)
C14A	0.0163 (18)	0.0170 (18)	0.022 (2)	−0.0011 (15)	0.0027 (16)	−0.0031 (15)
C15A	0.019 (2)	0.0198 (19)	0.023 (2)	0.0026 (15)	−0.0001 (17)	−0.0015 (15)
C16A	0.0158 (18)	0.0182 (19)	0.022 (2)	−0.0024 (15)	0.0006 (16)	−0.0021 (15)
C17A	0.0194 (19)	0.0178 (19)	0.027 (2)	0.0054 (15)	0.0023 (17)	0.0062 (16)
C18A	0.0131 (18)	0.0235 (19)	0.021 (2)	−0.0068 (15)	0.0010 (16)	−0.0002 (15)
C19A	0.0185 (19)	0.0178 (18)	0.021 (2)	0.0000 (15)	−0.0004 (16)	0.0040 (15)
C20A	0.0170 (18)	0.0167 (18)	0.0174 (19)	−0.0019 (14)	0.0002 (16)	0.0061 (14)
C21A	0.020 (2)	0.0133 (18)	0.026 (2)	0.0030 (15)	−0.0030 (17)	0.0094 (15)
C22A	0.0118 (18)	0.0241 (19)	0.0175 (19)	−0.0045 (15)	0.0033 (16)	0.0035 (15)
C23A	0.0171 (19)	0.026 (2)	0.023 (2)	−0.0039 (16)	0.0070 (17)	−0.0033 (16)
C24A	0.0153 (18)	0.0179 (19)	0.028 (2)	0.0008 (15)	−0.0042 (17)	0.0071 (15)
C25A	0.0122 (17)	0.0242 (19)	0.0132 (18)	−0.0039 (15)	0.0001 (15)	0.0028 (14)
C26A	0.0124 (17)	0.0234 (19)	0.0137 (18)	−0.0019 (15)	−0.0014 (15)	−0.0004 (14)
C27A	0.0126 (18)	0.0219 (19)	0.0125 (17)	−0.0021 (14)	−0.0031 (15)	0.0019 (14)
C28A	0.0150 (19)	0.029 (2)	0.0167 (19)	−0.0025 (16)	0.0019 (16)	−0.0005 (15)
C29A	0.0142 (18)	0.025 (2)	0.0177 (19)	0.0034 (15)	0.0019 (16)	−0.0038 (15)
C30A	0.0110 (17)	0.0176 (18)	0.0195 (19)	−0.0026 (14)	−0.0028 (15)	−0.0019 (14)
C31A	0.0130 (17)	0.0153 (18)	0.0214 (19)	0.0008 (14)	−0.0023 (16)	−0.0031 (14)
C32A	0.0146 (18)	0.0123 (17)	0.0205 (19)	0.0002 (14)	−0.0021 (16)	−0.0024 (14)
C33A	0.0126 (17)	0.0103 (17)	0.023 (2)	−0.0024 (14)	−0.0039 (16)	−0.0017 (14)
C34A	0.019 (2)	0.0172 (18)	0.022 (2)	0.0062 (15)	−0.0017 (17)	−0.0047 (15)
C35A	0.0158 (18)	0.0202 (19)	0.022 (2)	0.0058 (15)	−0.0027 (16)	0.0002 (15)
C36A	0.0109 (17)	0.0207 (19)	0.023 (2)	−0.0019 (14)	−0.0013 (16)	0.0003 (15)
O16B	0.039 (2)	0.116 (3)	0.062 (3)	0.015 (2)	0.004 (2)	0.026 (2)
O17B	0.063 (2)	0.0304 (19)	0.120 (4)	−0.0052 (17)	0.014 (2)	0.015 (2)
O18B	0.071 (3)	0.061 (2)	0.090 (3)	0.005 (2)	0.000 (2)	0.012 (2)
N2B	0.037 (2)	0.033 (2)	0.074 (3)	0.0101 (19)	0.008 (2)	0.005 (2)
O19B	0.0298 (15)	0.0346 (16)	0.0304 (16)	−0.0038 (13)	0.0062 (13)	0.0059 (12)
O20B	0.058 (2)	0.0225 (16)	0.047 (2)	0.0055 (14)	0.0009 (17)	−0.0018 (13)
O21B	0.0202 (14)	0.0467 (18)	0.0298 (16)	0.0010 (13)	0.0048 (13)	−0.0004 (13)
N3B	0.0278 (19)	0.032 (2)	0.0149 (16)	−0.0007 (15)	−0.0062 (15)	0.0015 (14)
O19A	0.0395 (18)	0.0464 (18)	0.0409 (19)	−0.0217 (15)	−0.0048 (15)	0.0045 (15)
O20A	0.059 (2)	0.0227 (16)	0.0452 (19)	−0.0012 (14)	−0.0025 (17)	−0.0048 (13)
O21A	0.0336 (18)	0.0471 (19)	0.056 (2)	0.0099 (15)	0.0060 (16)	−0.0039 (16)
N3A	0.0308 (19)	0.0277 (19)	0.0186 (17)	−0.0026 (15)	0.0007 (15)	−0.0011 (14)
O16A	0.0315 (18)	0.066 (2)	0.074 (3)	−0.0070 (16)	−0.0174 (19)	0.0213 (19)
O17A	0.072 (3)	0.051 (2)	0.073 (3)	−0.005 (2)	0.005 (2)	−0.0041 (19)
O18A	0.0312 (17)	0.063 (2)	0.0327 (18)	0.0064 (16)	0.0012 (14)	0.0125 (15)
N2A	0.0233 (19)	0.053 (2)	0.033 (2)	−0.0084 (18)	0.0037 (17)	0.0140 (18)
O15W	0.0251 (15)	0.0420 (17)	0.0403 (18)	−0.0014 (13)	−0.0071 (14)	−0.0045 (14)
O1W	0.0324 (16)	0.0225 (14)	0.0418 (18)	0.0032 (12)	0.0070 (14)	0.0011 (12)
O17W	0.033 (3)	0.094 (5)	0.124 (6)	−0.009 (3)	0.005 (3)	0.046 (4)
O3W	0.0174 (14)	0.0448 (17)	0.0361 (17)	0.0048 (12)	−0.0038 (13)	0.0002 (13)
O23W	0.038 (7)	0.111 (9)	0.154 (13)	0.011 (7)	0.007 (10)	0.013 (11)
O2W	0.0188 (13)	0.0245 (14)	0.0353 (16)	−0.0014 (11)	−0.0041 (12)	0.0022 (12)
O21W	0.070 (6)	0.066 (4)	0.069 (6)	−0.020 (4)	0.024 (5)	−0.012 (4)
O19W	0.059 (2)	0.060 (2)	0.081 (3)	−0.0130 (18)	0.036 (2)	−0.013 (2)
O14W	0.0380 (19)	0.085 (3)	0.049 (2)	0.0254 (17)	−0.0152 (17)	−0.0354 (19)
O6W	0.054 (3)	0.102 (3)	0.083 (3)	−0.006 (3)	−0.017 (2)	0.024 (3)
O16W	0.0255 (15)	0.0321 (16)	0.0445 (18)	−0.0042 (12)	0.0037 (14)	−0.0033 (13)
O4W	0.0185 (14)	0.0427 (17)	0.0325 (16)	0.0001 (12)	−0.0091 (13)	0.0033 (13)
O18W	0.028 (8)	0.094 (10)	0.134 (11)	−0.007 (8)	0.021 (9)	0.045 (9)
O5W	0.056 (8)	0.040 (7)	0.035 (7)	−0.008 (6)	0.011 (6)	−0.007 (5)
O10W	0.0260 (15)	0.0349 (16)	0.0359 (17)	−0.0024 (12)	−0.0043 (13)	0.0050 (13)
O20W	0.082 (11)	0.070 (7)	0.076 (11)	−0.003 (8)	0.019 (8)	−0.026 (8)
O11W	0.0243 (14)	0.0309 (15)	0.0264 (15)	0.0040 (12)	0.0037 (12)	0.0001 (12)
O22W	0.032 (4)	0.116 (6)	0.160 (11)	−0.011 (4)	−0.011 (6)	0.072 (7)
O12W	0.0298 (16)	0.0336 (16)	0.051 (2)	0.0051 (13)	0.0021 (15)	0.0091 (14)
O25W	0.054 (2)	0.160 (4)	0.053 (3)	−0.008 (3)	0.005 (2)	0.026 (3)
O13W	0.0425 (19)	0.0503 (19)	0.052 (2)	−0.0060 (15)	−0.0046 (17)	0.0181 (16)
O24W	0.0298 (18)	0.056 (2)	0.106 (3)	−0.0114 (16)	−0.006 (2)	0.014 (2)

### Pentaaqua(cucurbit[6]uril-κ2O,O')(nitrato-κ2O,O')praseodymium(III) dinitrate 9.56-hydrate Geometric parameters (Å, º)

**Table:**

Pr1A—O1WA	2.544 (2)	Pr1B—O1WB	2.553 (3)
Pr1A—O2WA	2.449 (3)	Pr1B—O2WB	2.438 (2)
Pr1A—O3WA	2.442 (2)	Pr1B—O3WB	2.448 (2)
Pr1B—O4WB	2.527 (3)	Pr1B—O4WB	2.527 (3)
Pr1A—O5A	2.493 (2)	Pr1B—O5B	2.519 (2)
Pr1A—O5WA	2.483 (2)	Pr1B—O5WB	2.476 (2)
Pr1A—O6A	2.466 (2)	Pr1B—O6B	2.460 (2)
Pr1A—O13A	2.571 (2)	Pr1B—O13B	2.651 (3)
Pr1A—O14A	2.613 (2)	Pr1B—O14B	2.572 (2)
			
O2WA—Pr1A—O1WA	68.01 (9)	O2WB—Pr1B—O13B	68.61 (8)
O2WA—Pr1A—O4WA	77.25 (8)	O2WB—Pr1B—O3WB	84.55 (8)
O2WA—Pr1A—O5WA	67.92 (8)	O2WB—Pr1B—O6B	96.37 (8)
O2WA—Pr1A—O6A	84.64 (8)	O2WB—Pr1B—O1WB	67.77 (9)
O3WA—Pr1A—O1WA	73.57 (9)	O2WB—Pr1B—O5WB	71.46 (8)
O3WA—Pr1A—O2WA	93.13 (9)	O3WB—Pr1B—O5WB	71.21 (8)
O3WA—Pr1A—O5WA	70.71 (8)	O3WB—Pr1B—O5B	78.97 (8)
O3WA—Pr1A—O5A	77.35 (8)	O3WB—Pr1B—O4WB	76.07 (9)
O3WA—Pr1A—O13A	73.92 (8)	O3WB—Pr1B—O1WB	71.69 (9)
O4WA—Pr1A—O13A	88.91 (8)	O3WB—Pr1B—O1WB	71.69 (9)
O4WA—Pr1A—O14A	65.33 (8)	O4WB—Pr1B—O14B	65.36 (8)
O5WA—Pr1A—O4WA	71.24 (8)	O5B—Pr1B—O4WB	70.75 (8)
O5WA—Pr1A—O13A	70.72 (8)	O5B—Pr1B—O1WB	71.12 (9)
O5A—Pr1A—O1WA	72.17 (8)	O5WB—Pr1B—O4WB	69.88 (8)
O5A—Pr1A—O13A	76.26 (8)	O5WB—Pr1B—O14B	82.62 (8)
O5A—Pr1A—O14A	71.00 (8)	O5WB—Pr1B—O13B	71.53 (8)
O6A—Pr1A—O1WA	74.26 (8)	O6B—Pr1B—O1WB	75.85 (9)
O6A—Pr1A—O4WA	70.85 (8)	O6B—Pr1B—O5B	77.22 (8)
O6A—Pr1A—O5A	82.86 (8)	O6B—Pr1B—O14B	71.49 (8)
O6A—Pr1A—O14A	78.55 (7)	O6B—Pr1B—O13B	71.15 (8)
O13A—Pr1A—O14A	49.19 (7)	O14B—Pr1B—O13B	48.49 (8)
			
Pr1B—O13B—N1B—O15B	−171.1 (3)	Pr1A—O14A—N1A—O15A	177.0 (3)
Pr1B—O13B—N1B—O14B	6.9 (3)	Pr1A—O14A—N1A—O13A	−1.7 (3)
Pr1B—O14B—N1B—O15B	170.8 (2)	Pr1A—O13A—N1A—O15A	−177.0 (3)
Pr1B—O14B—N1B—O13B	−7.1 (3)	Pr1A—O13A—N1A—O14A	1.7 (3)
C4B—N5B—C1B—N4B	110.1 (3)	C4A—N5A—C1A—N4A	−108.5 (3)
C35B—N5B—C1B—N4B	−85.0 (4)	C35A—N5A—C1A—N4A	79.6 (4)
C4B—N5B—C1B—C2B	−1.6 (3)	C4A—N5A—C1A—C2A	3.1 (4)
C35B—N5B—C1B—C2B	163.2 (3)	C35A—N5A—C1A—C2A	−168.9 (3)
C3B—N4B—C1B—N5B	−116.9 (3)	C3A—N4A—C1A—N5A	118.6 (3)
C36B—N4B—C1B—N5B	65.3 (4)	C36A—N4A—C1A—N5A	−68.1 (4)
C3B—N4B—C1B—C2B	−4.6 (3)	C3A—N4A—C1A—C2A	6.8 (4)
C36B—N4B—C1B—C2B	177.5 (3)	C36A—N4A—C1A—C2A	−179.9 (3)
C3B—N7B—C2B—N6B	104.4 (3)	C3A—N7A—C2A—N6A	−108.1 (3)
C6B—N7B—C2B—N6B	−67.2 (4)	C6A—N7A—C2A—N6A	67.4 (4)
C3B—N7B—C2B—C1B	−6.0 (4)	C3A—N7A—C2A—C1A	3.2 (4)
C6B—N7B—C2B—C1B	−177.6 (3)	C6A—N7A—C2A—C1A	178.7 (3)
C4B—N6B—C2B—N7B	−117.7 (3)	C4A—N6A—C2A—N7A	118.9 (3)
C5B—N6B—C2B—N7B	82.5 (4)	C5A—N6A—C2A—N7A	−84.5 (4)
C4B—N6B—C2B—C1B	−7.0 (3)	C4A—N6A—C2A—C1A	7.6 (4)
C5B—N6B—C2B—C1B	−166.8 (3)	C5A—N6A—C2A—C1A	164.2 (3)
N5B—C1B—C2B—N7B	125.2 (3)	N5A—C1A—C2A—N7A	−126.2 (3)
N4B—C1B—C2B—N7B	6.0 (3)	N4A—C1A—C2A—N7A	−5.7 (3)
N5B—C1B—C2B—N6B	4.9 (3)	N5A—C1A—C2A—N6A	−6.1 (3)
N4B—C1B—C2B—N6B	−114.2 (3)	N4A—C1A—C2A—N6A	114.3 (3)
C2B—N7B—C3B—O1B	−179.6 (3)	C36A—N4A—C3A—O1A	−3.0 (5)
C6B—N7B—C3B—O1B	−7.6 (5)	C1A—N4A—C3A—O1A	170.5 (3)
C2B—N7B—C3B—N4B	3.4 (4)	C36A—N4A—C3A—N7A	−178.6 (3)
C6B—N7B—C3B—N4B	175.4 (3)	C1A—N4A—C3A—N7A	−5.1 (4)
C36B—N4B—C3B—O1B	2.0 (5)	C6A—N7A—C3A—O1A	9.7 (5)
C1B—N4B—C3B—O1B	−175.9 (3)	C2A—N7A—C3A—O1A	−174.7 (3)
C36B—N4B—C3B—N7B	179.0 (3)	C6A—N7A—C3A—N4A	−174.8 (3)
C1B—N4B—C3B—N7B	1.1 (4)	C2A—N7A—C3A—N4A	0.9 (4)
C5B—N6B—C4B—O9B	−10.9 (5)	C5A—N6A—C4A—O9A	16.0 (6)
C2B—N6B—C4B—O9B	−171.4 (3)	C2A—N6A—C4A—O9A	173.4 (3)
C5B—N6B—C4B—N5B	166.8 (3)	C5A—N6A—C4A—N5A	−163.4 (3)
C2B—N6B—C4B—N5B	6.3 (4)	C2A—N6A—C4A—N5A	−6.0 (4)
C1B—N5B—C4B—O9B	175.1 (3)	C1A—N5A—C4A—O9A	−177.8 (3)
C35B—N5B—C4B—O9B	10.0 (5)	C35A—N5A—C4A—O9A	−5.8 (6)
C1B—N5B—C4B—N6B	−2.7 (4)	C1A—N5A—C4A—N6A	1.6 (4)
C35B—N5B—C4B—N6B	−167.8 (3)	C35A—N5A—C4A—N6A	173.7 (3)
C10B—N9B—C5B—N6B	−110.6 (4)	C10A—N9A—C5A—N6A	111.7 (4)
C7B—N9B—C5B—N6B	79.2 (4)	C7A—N9A—C5A—N6A	−79.4 (4)
C4B—N6B—C5B—N9B	108.8 (3)	C4A—N6A—C5A—N9A	−111.3 (4)
C2B—N6B—C5B—N9B	−93.0 (4)	C2A—N6A—C5A—N9A	94.1 (4)
C9B—N8B—C6B—N7B	107.3 (4)	C9A—N8A—C6A—N7A	−106.1 (4)
C7B—N8B—C6B—N7B	−94.4 (4)	C7A—N8A—C6A—N7A	93.4 (4)
C3B—N7B—C6B—N8B	−94.6 (4)	C3A—N7A—C6A—N8A	100.6 (4)
C2B—N7B—C6B—N8B	76.4 (4)	C2A—N7A—C6A—N8A	−74.5 (4)
C9B—N8B—C7B—N9B	−111.9 (3)	C10A—N9A—C7A—N8A	−116.5 (3)
C6B—N8B—C7B—N9B	87.9 (4)	C5A—N9A—C7A—N8A	73.4 (4)
C9B—N8B—C7B—C8B	−0.8 (4)	C10A—N9A—C7A—C8A	−5.2 (4)
C6B—N8B—C7B—C8B	−160.9 (3)	C5A—N9A—C7A—C8A	−175.3 (3)
C10B—N9B—C7B—N8B	116.1 (3)	C9A—N8A—C7A—N9A	109.0 (3)
C5B—N9B—C7B—N8B	−72.8 (4)	C6A—N8A—C7A—N9A	−88.8 (4)
C10B—N9B—C7B—C8B	4.4 (4)	C9A—N8A—C7A—C8A	−2.2 (4)
C5B—N9B—C7B—C8B	175.6 (3)	C6A—N8A—C7A—C8A	159.9 (3)
C10B—N10B—C8B—N11B	−110.4 (3)	C10A—N10A—C8A—N11A	110.6 (3)
C11B—N10B—C8B—N11B	81.1 (4)	C11A—N10A—C8A—N11A	−80.3 (4)
C10B—N10B—C8B—C7B	1.1 (4)	C10A—N10A—C8A—C7A	−1.0 (4)
C11B—N10B—C8B—C7B	−167.5 (3)	C11A—N10A—C8A—C7A	168.1 (3)
C9B—N11B—C8B—N10B	118.2 (3)	C9A—N11A—C8A—N10A	−113.7 (3)
C12B—N11B—C8B—N10B	−78.8 (4)	C12A—N11A—C8A—N10A	77.3 (4)
C9B—N11B—C8B—C7B	6.4 (4)	C9A—N11A—C8A—C7A	−2.0 (4)
C12B—N11B—C8B—C7B	169.4 (3)	C12A—N11A—C8A—C7A	−170.9 (3)
N8B—C7B—C8B—N10B	−122.6 (3)	N9A—C7A—C8A—N10A	3.5 (3)
N9B—C7B—C8B—N10B	−3.1 (3)	N8A—C7A—C8A—N10A	122.3 (3)
N8B—C7B—C8B—N11B	−3.3 (3)	N9A—C7A—C8A—N11A	−116.4 (3)
N9B—C7B—C8B—N11B	116.2 (3)	N8A—C7A—C8A—N11A	2.4 (3)
C6B—N8B—C9B—O2B	−14.3 (6)	C12A—N11A—C9A—O2A	−9.4 (6)
C7B—N8B—C9B—O2B	−174.5 (4)	C8A—N11A—C9A—O2A	−178.2 (4)
C6B—N8B—C9B—N11B	165.0 (3)	C12A—N11A—C9A—N8A	169.5 (3)
C7B—N8B—C9B—N11B	4.8 (4)	C8A—N11A—C9A—N8A	0.7 (4)
C12B—N11B—C9B—O2B	9.1 (6)	C6A—N8A—C9A—O2A	17.7 (6)
C8B—N11B—C9B—O2B	172.1 (4)	C7A—N8A—C9A—O2A	180.0 (4)
C12B—N11B—C9B—N8B	−170.1 (3)	C6A—N8A—C9A—N11A	−161.2 (3)
C8B—N11B—C9B—N8B	−7.2 (4)	C7A—N8A—C9A—N11A	1.1 (4)
C5B—N9B—C10B—O10B	4.6 (6)	C5A—N9A—C10A—O10A	−4.9 (6)
C7B—N9B—C10B—O10B	175.7 (4)	C7A—N9A—C10A—O10A	−174.8 (3)
C5B—N9B—C10B—N10B	−175.0 (3)	C5A—N9A—C10A—N10A	174.7 (3)
C7B—N9B—C10B—N10B	−3.9 (4)	C7A—N9A—C10A—N10A	4.8 (4)
C11B—N10B—C10B—O10B	−9.4 (6)	C8A—N10A—C10A—O10A	177.4 (3)
C8B—N10B—C10B—O10B	−178.0 (3)	C11A—N10A—C10A—O10A	8.2 (6)
C11B—N10B—C10B—N9B	170.2 (3)	C8A—N10A—C10A—N9A	−2.2 (4)
C8B—N10B—C10B—N9B	1.6 (4)	C11A—N10A—C10A—N9A	−171.4 (3)
C10B—N10B—C11B—N12B	105.6 (4)	C16A—N12A—C11A—N10A	93.6 (4)
C8B—N10B—C11B—N12B	−87.0 (4)	C13A—N12A—C11A—N10A	−82.1 (4)
C16B—N12B—C11B—N10B	−101.7 (4)	C10A—N10A—C11A—N12A	−105.1 (4)
C13B—N12B—C11B—N10B	82.6 (4)	C8A—N10A—C11A—N12A	87.0 (4)
C9B—N11B—C12B—N13B	−112.7 (4)	C15A—N13A—C12A—N11A	−115.7 (3)
C8B—N11B—C12B—N13B	86.1 (4)	C13A—N13A—C12A—N11A	89.9 (4)
C15B—N13B—C12B—N11B	108.2 (4)	C9A—N11A—C12A—N13A	105.9 (4)
C13B—N13B—C12B—N11B	−88.0 (4)	C8A—N11A—C12A—N13A	−86.3 (4)
C15B—N13B—C13B—N12B	−115.3 (3)	C16A—N12A—C13A—N13A	−101.8 (3)
C12B—N13B—C13B—N12B	79.4 (4)	C11A—N12A—C13A—N13A	74.3 (4)
C15B—N13B—C13B—C14B	−4.2 (4)	C16A—N12A—C13A—C14A	9.5 (4)
C12B—N13B—C13B—C14B	−169.5 (3)	C11A—N12A—C13A—C14A	−174.4 (3)
C16B—N12B—C13B—N13B	107.9 (3)	C15A—N13A—C13A—N12A	124.0 (3)
C11B—N12B—C13B—N13B	−76.0 (4)	C12A—N13A—C13A—N12A	−79.4 (4)
C16B—N12B—C13B—C14B	−3.2 (3)	C15A—N13A—C13A—C14A	12.7 (4)
C11B—N12B—C13B—C14B	172.9 (3)	C12A—N13A—C13A—C14A	169.2 (3)
C16B—N15B—C14B—N14B	−116.4 (3)	C15A—N14A—C14A—N15A	−103.3 (4)
C18B—N15B—C14B—N14B	68.0 (4)	C17A—N14A—C14A—N15A	68.5 (4)
C16B—N15B—C14B—C13B	−4.8 (4)	C15A—N14A—C14A—C13A	8.0 (4)
C18B—N15B—C14B—C13B	179.6 (3)	C17A—N14A—C14A—C13A	179.7 (3)
C15B—N14B—C14B—N15B	107.6 (3)	C16A—N15A—C14A—N14A	122.4 (3)
C17B—N14B—C14B—N15B	−78.4 (4)	C18A—N15A—C14A—N14A	−78.7 (4)
C15B—N14B—C14B—C13B	−4.0 (4)	C16A—N15A—C14A—C13A	11.1 (4)
C17B—N14B—C14B—C13B	169.9 (3)	C18A—N15A—C14A—C13A	170.0 (3)
N13B—C13B—C14B—N15B	−115.4 (3)	N12A—C13A—C14A—N14A	−132.1 (3)
N12B—C13B—C14B—N15B	4.6 (3)	N13A—C13A—C14A—N14A	−11.9 (3)
N13B—C13B—C14B—N14B	4.7 (3)	N12A—C13A—C14A—N15A	−11.7 (3)
N12B—C13B—C14B—N14B	124.6 (3)	N13A—C13A—C14A—N15A	108.5 (3)
C13B—N13B—C15B—O3B	−176.1 (3)	C17A—N14A—C15A—O3A	10.3 (6)
C12B—N13B—C15B—O3B	−10.8 (5)	C14A—N14A—C15A—O3A	−177.9 (3)
C13B—N13B—C15B—N14B	1.9 (4)	C17A—N14A—C15A—N13A	−172.2 (3)
C12B—N13B—C15B—N14B	167.2 (3)	C14A—N14A—C15A—N13A	−0.4 (4)
C17B—N14B—C15B—O3B	5.6 (6)	C12A—N13A—C15A—O3A	12.2 (5)
C14B—N14B—C15B—O3B	179.6 (3)	C13A—N13A—C15A—O3A	169.2 (3)
C17B—N14B—C15B—N13B	−172.4 (3)	C12A—N13A—C15A—N14A	−165.3 (3)
C14B—N14B—C15B—N13B	1.6 (4)	C13A—N13A—C15A—N14A	−8.3 (4)
C14B—N15B—C16B—O11B	−174.3 (3)	C13A—N12A—C16A—O11A	178.8 (3)
C18B—N15B—C16B—O11B	1.4 (6)	C11A—N12A—C16A—O11A	2.5 (5)
C14B—N15B—C16B—N12B	2.9 (4)	C13A—N12A—C16A—N15A	−2.9 (4)
C18B—N15B—C16B—N12B	178.6 (3)	C11A—N12A—C16A—N15A	−179.2 (3)
C11B—N12B—C16B—O11B	1.5 (5)	C14A—N15A—C16A—O11A	172.6 (3)
C13B—N12B—C16B—O11B	177.7 (3)	C18A—N15A—C16A—O11A	13.2 (5)
C11B—N12B—C16B—N15B	−175.7 (3)	C14A—N15A—C16A—N12A	−5.7 (4)
C13B—N12B—C16B—N15B	0.5 (4)	C18A—N15A—C16A—N12A	−165.1 (3)
C15B—N14B—C17B—N17B	−91.9 (4)	C15A—N14A—C17A—N17A	88.9 (4)
C14B—N14B—C17B—N17B	94.7 (4)	C14A—N14A—C17A—N17A	−82.0 (4)
C21B—N17B—C17B—N14B	92.7 (4)	C21A—N17A—C17A—N14A	−101.1 (4)
C19B—N17B—C17B—N14B	−81.9 (4)	C19A—N17A—C17A—N14A	94.4 (4)
C16B—N15B—C18B—N16B	100.9 (4)	C22A—N16A—C18A—N15A	96.8 (4)
C14B—N15B—C18B—N16B	−83.9 (4)	C19A—N16A—C18A—N15A	−82.5 (4)
C22B—N16B—C18B—N15B	−100.7 (4)	C16A—N15A—C18A—N16A	−109.6 (4)
C19B—N16B—C18B—N15B	95.7 (4)	C14A—N15A—C18A—N16A	93.4 (4)
C21B—N17B—C19B—N16B	−109.0 (3)	C22A—N16A—C19A—N17A	−110.8 (3)
C17B—N17B—C19B—N16B	66.0 (4)	C18A—N16A—C19A—N17A	68.6 (4)
C21B—N17B—C19B—C20B	1.8 (4)	C22A—N16A—C19A—C20A	1.0 (4)
C17B—N17B—C19B—C20B	176.8 (3)	C18A—N16A—C19A—C20A	−179.6 (3)
C22B—N16B—C19B—N17B	116.9 (3)	C21A—N17A—C19A—N16A	114.8 (3)
C18B—N16B—C19B—N17B	−78.1 (4)	C17A—N17A—C19A—N16A	−79.4 (4)
C22B—N16B—C19B—C20B	5.8 (4)	C21A—N17A—C19A—C20A	3.3 (4)
C18B—N16B—C19B—C20B	170.9 (3)	C17A—N17A—C19A—C20A	169.1 (3)
C22B—N18B—C20B—N19B	−108.2 (3)	C22A—N18A—C20A—N19A	112.4 (3)
C23B—N18B—C20B—N19B	82.1 (4)	C23A—N18A—C20A—N19A	−82.5 (4)
C22B—N18B—C20B—C19B	3.1 (4)	C22A—N18A—C20A—C19A	1.5 (4)
C23B—N18B—C20B—C19B	−166.5 (3)	C23A—N18A—C20A—C19A	166.6 (3)
C21B—N19B—C20B—N18B	119.1 (3)	C21A—N19A—C20A—N18A	−114.1 (3)
C24B—N19B—C20B—N18B	−76.9 (4)	C24A—N19A—C20A—N18A	77.8 (4)
C21B—N19B—C20B—C19B	7.4 (4)	C21A—N19A—C20A—C19A	−2.9 (4)
C24B—N19B—C20B—C19B	171.4 (3)	C24A—N19A—C20A—C19A	−171.0 (3)
N17B—C19B—C20B—N18B	−124.9 (3)	N16A—C19A—C20A—N18A	−1.4 (3)
N16B—C19B—C20B—N18B	−5.1 (3)	N17A—C19A—C20A—N18A	118.6 (3)
N17B—C19B—C20B—N19B	−5.3 (3)	N16A—C19A—C20A—N19A	−120.3 (3)
N16B—C19B—C20B—N19B	114.5 (3)	N17A—C19A—C20A—N19A	−0.2 (3)
C24B—N19B—C21B—O4B	5.6 (5)	C19A—N17A—C21A—O4A	173.6 (3)
C20B—N19B—C21B—O4B	169.8 (3)	C17A—N17A—C21A—O4A	7.6 (5)
C24B—N19B—C21B—N17B	−170.8 (3)	C19A—N17A—C21A—N19A	−5.3 (4)
C20B—N19B—C21B—N17B	−6.6 (4)	C17A—N17A—C21A—N19A	−171.4 (3)
C17B—N17B—C21B—O4B	11.2 (5)	C24A—N19A—C21A—O4A	−5.5 (5)
C19B—N17B—C21B—O4B	−173.7 (3)	C20A—N19A—C21A—O4A	−173.8 (3)
C17B—N17B—C21B—N19B	−172.3 (3)	C24A—N19A—C21A—N17A	173.4 (3)
C19B—N17B—C21B—N19B	2.8 (4)	C20A—N19A—C21A—N17A	5.1 (4)
C23B—N18B—C22B—O12B	−8.8 (6)	C19A—N16A—C22A—O12A	−178.9 (3)
C20B—N18B—C22B—O12B	−178.6 (3)	C18A—N16A—C22A—O12A	1.7 (5)
C23B—N18B—C22B—N16B	170.2 (3)	C19A—N16A—C22A—N18A	−0.1 (4)
C20B—N18B—C22B—N16B	0.4 (4)	C18A—N16A—C22A—N18A	−179.5 (3)
C18B—N16B—C22B—O12B	9.6 (6)	C20A—N18A—C22A—O12A	177.8 (3)
C19B—N16B—C22B—O12B	174.8 (3)	C23A—N18A—C22A—O12A	12.6 (5)
C18B—N16B—C22B—N18B	−169.4 (3)	C20A—N18A—C22A—N16A	−0.9 (4)
C19B—N16B—C22B—N18B	−4.2 (4)	C23A—N18A—C22A—N16A	−166.1 (3)
C22B—N18B—C23B—N20B	103.4 (4)	C28A—N20A—C23A—N18A	109.9 (4)
C20B—N18B—C23B—N20B	−88.1 (4)	C25A—N20A—C23A—N18A	−83.9 (4)
C28B—N20B—C23B—N18B	−108.8 (4)	C22A—N18A—C23A—N20A	−107.5 (4)
C25B—N20B—C23B—N18B	83.7 (4)	C20A—N18A—C23A—N20A	88.9 (4)
C21B—N19B—C24B—N21B	−114.2 (3)	C21A—N19A—C24A—N21A	109.7 (4)
C20B—N19B—C24B—N21B	83.4 (4)	C20A—N19A—C24A—N21A	−83.4 (4)
C27B—N21B—C24B—N19B	116.2 (3)	C27A—N21A—C24A—N19A	−109.3 (4)
C25B—N21B—C24B—N19B	−89.3 (4)	C25A—N21A—C24A—N19A	86.9 (4)
C28B—N20B—C25B—N21B	110.8 (3)	C28A—N20A—C25A—N21A	−114.8 (3)
C23B—N20B—C25B—N21B	−80.5 (4)	C23A—N20A—C25A—N21A	77.7 (4)
C28B—N20B—C25B—C26B	−0.6 (4)	C28A—N20A—C25A—C26A	−3.2 (4)
C23B—N20B—C25B—C26B	168.1 (3)	C23A—N20A—C25A—C26A	−170.7 (3)
C27B—N21B—C25B—N20B	−117.5 (3)	C27A—N21A—C25A—N20A	113.5 (3)
C24B—N21B—C25B—N20B	85.3 (4)	C24A—N21A—C25A—N20A	−81.1 (4)
C27B—N21B—C25B—C26B	−6.3 (4)	C27A—N21A—C25A—C26A	1.8 (4)
C24B—N21B—C25B—C26B	−163.4 (3)	C24A—N21A—C25A—C26A	167.2 (3)
C27B—N23B—C26B—N22B	108.9 (3)	C27A—N23A—C26A—N22A	−111.0 (3)
C30B—N23B—C26B—N22B	−75.4 (4)	C30A—N23A—C26A—N22A	78.4 (4)
C27B—N23B—C26B—C25B	−2.6 (4)	C27A—N23A—C26A—C25A	0.8 (4)
C30B—N23B—C26B—C25B	173.1 (3)	C30A—N23A—C26A—C25A	−169.8 (3)
C28B—N22B—C26B—N23B	−119.8 (3)	C28A—N22A—C26A—N23A	118.3 (3)
C29B—N22B—C26B—N23B	83.6 (4)	C29A—N22A—C26A—N23A	−76.4 (4)
C28B—N22B—C26B—C25B	−8.7 (4)	C28A—N22A—C26A—C25A	6.8 (4)
C29B—N22B—C26B—C25B	−165.2 (3)	C29A—N22A—C26A—C25A	172.1 (3)
N20B—C25B—C26B—N23B	124.2 (3)	N20A—C25A—C26A—N23A	−121.4 (3)
N21B—C25B—C26B—N23B	5.1 (3)	N21A—C25A—C26A—N23A	−1.5 (3)
N20B—C25B—C26B—N22B	5.4 (3)	N20A—C25A—C26A—N22A	−2.0 (3)
N21B—C25B—C26B—N22B	−113.6 (3)	N21A—C25A—C26A—N22A	117.9 (3)
Pr1B—O5B—C27B—N23B	54.7 (6)	Pr1A—O5A—C27A—N23A	−28.3 (7)
Pr1B—O5B—C27B—N21B	−126.2 (4)	Pr1A—O5A—C27A—N21A	152.9 (3)
C30B—N23B—C27B—O5B	2.4 (5)	C30A—N23A—C27A—O5A	−8.2 (5)
C26B—N23B—C27B—O5B	178.0 (3)	C26A—N23A—C27A—O5A	−178.6 (3)
C30B—N23B—C27B—N21B	−176.8 (3)	C30A—N23A—C27A—N21A	170.8 (3)
C26B—N23B—C27B—N21B	−1.2 (4)	C26A—N23A—C27A—N21A	0.3 (4)
C24B—N21B—C27B—O5B	−17.6 (5)	C24A—N21A—C27A—O5A	12.2 (5)
C25B—N21B—C27B—O5B	−174.3 (3)	C25A—N21A—C27A—O5A	177.6 (3)
C24B—N21B—C27B—N23B	161.6 (3)	C24A—N21A—C27A—N23A	−166.8 (3)
C25B—N21B—C27B—N23B	4.9 (4)	C25A—N21A—C27A—N23A	−1.4 (4)
C25B—N20B—C28B—O7B	174.6 (3)	C29A—N22A—C28A—O7A	4.6 (6)
C23B—N20B—C28B—O7B	6.0 (6)	C26A—N22A—C28A—O7A	170.1 (4)
C25B—N20B—C28B—N22B	−4.8 (4)	C29A—N22A—C28A—N20A	−174.5 (3)
C23B—N20B—C28B—N22B	−173.4 (3)	C26A—N22A—C28A—N20A	−9.0 (4)
C29B—N22B—C28B—O7B	−14.2 (6)	C25A—N20A—C28A—O7A	−171.6 (4)
C26B—N22B—C28B—O7B	−170.7 (4)	C23A—N20A—C28A—O7A	−3.9 (6)
C29B—N22B—C28B—N20B	165.2 (3)	C25A—N20A—C28A—N22A	7.6 (4)
C26B—N22B—C28B—N20B	8.7 (4)	C23A—N20A—C28A—N22A	175.2 (3)
C34B—N24B—C29B—N22B	−104.7 (4)	C28A—N22A—C29A—N24A	−110.0 (4)
C31B—N24B—C29B—N22B	82.0 (4)	C26A—N22A—C29A—N24A	86.2 (4)
C28B—N22B—C29B—N24B	112.1 (4)	C34A—N24A—C29A—N22A	111.2 (3)
C26B—N22B—C29B—N24B	−93.9 (4)	C31A—N24A—C29A—N22A	−88.7 (4)
C27B—N23B—C30B—N25B	−100.6 (4)	C27A—N23A—C30A—N25A	100.4 (4)
C26B—N23B—C30B—N25B	84.2 (4)	C26A—N23A—C30A—N25A	−90.1 (4)
C33B—N25B—C30B—N23B	90.8 (4)	C33A—N25A—C30A—N23A	−98.8 (4)
C31B—N25B—C30B—N23B	−91.8 (4)	C31A—N25A—C30A—N23A	88.1 (4)
C33B—N25B—C31B—N24B	−104.3 (3)	C33A—N25A—C31A—N24A	109.5 (3)
C30B—N25B—C31B—N24B	78.1 (4)	C30A—N25A—C31A—N24A	−76.7 (4)
C33B—N25B—C31B—C32B	6.2 (4)	C33A—N25A—C31A—C32A	−1.7 (3)
C30B—N25B—C31B—C32B	−171.4 (3)	C30A—N25A—C31A—C32A	172.1 (3)
C34B—N24B—C31B—N25B	117.5 (3)	C34A—N24A—C31A—N25A	−119.0 (3)
C29B—N24B—C31B—N25B	−68.6 (4)	C29A—N24A—C31A—N25A	79.1 (4)
C34B—N24B—C31B—C32B	7.1 (4)	C34A—N24A—C31A—C32A	−8.3 (4)
C29B—N24B—C31B—C32B	−179.0 (3)	C29A—N24A—C31A—C32A	−170.2 (3)
C34B—N26B—C32B—N27B	−108.0 (3)	C34A—N26A—C32A—N27A	111.7 (3)
C35B—N26B—C32B—N27B	69.7 (4)	C35A—N26A—C32A—N27A	−71.1 (4)
C34B—N26B—C32B—C31B	3.2 (4)	C34A—N26A—C32A—C31A	−0.3 (3)
C35B—N26B—C32B—C31B	−179.1 (3)	C35A—N26A—C32A—C31A	176.9 (3)
C33B—N27B—C32B—N26B	117.4 (3)	C33A—N27A—C32A—N26A	−116.2 (3)
C36B—N27B—C32B—N26B	−88.2 (4)	C36A—N27A—C32A—N26A	79.9 (4)
C33B—N27B—C32B—C31B	6.2 (4)	C33A—N27A—C32A—C31A	−4.4 (3)
C36B—N27B—C32B—C31B	160.6 (3)	C36A—N27A—C32A—C31A	−168.4 (3)
N25B—C31B—C32B—N26B	−125.6 (3)	N25A—C31A—C32A—N26A	123.7 (3)
N24B—C31B—C32B—N26B	−5.9 (3)	N24A—C31A—C32A—N26A	4.9 (3)
N25B—C31B—C32B—N27B	−7.1 (3)	N25A—C31A—C32A—N27A	3.5 (3)
N24B—C31B—C32B—N27B	112.6 (3)	N24A—C31A—C32A—N27A	−115.3 (3)
Pr1B—O6B—C33B—N27B	154.6 (6)	Pr1A—O6A—C33A—N25A	36.4 (7)
Pr1B—O6B—C33B—N25B	−25.9 (10)	Pr1A—O6A—C33A—N27A	−145.6 (4)
C32B—N27B—C33B—O6B	177.0 (3)	C31A—N25A—C33A—O6A	177.1 (3)
C36B—N27B—C33B—O6B	22.4 (5)	C30A—N25A—C33A—O6A	3.5 (5)
C32B—N27B—C33B—N25B	−2.6 (4)	C31A—N25A—C33A—N27A	−1.1 (4)
C36B—N27B—C33B—N25B	−157.1 (3)	C30A—N25A—C33A—N27A	−174.7 (3)
C31B—N25B—C33B—O6B	177.8 (3)	C36A—N27A—C33A—O6A	−10.6 (5)
C30B—N25B—C33B—O6B	−4.5 (5)	C32A—N27A—C33A—O6A	−174.6 (3)
C31B—N25B—C33B—N27B	−2.7 (4)	C36A—N27A—C33A—N25A	167.7 (3)
C30B—N25B—C33B—N27B	175.0 (3)	C32A—N27A—C33A—N25A	3.6 (4)
C35B—N26B—C34B—O8B	6.5 (5)	C29A—N24A—C34A—O8A	−7.8 (5)
C32B—N26B—C34B—O8B	−175.9 (3)	C31A—N24A—C34A—O8A	−170.1 (3)
C35B—N26B—C34B—N24B	−176.6 (3)	C29A—N24A—C34A—N26A	170.7 (3)
C32B—N26B—C34B—N24B	1.1 (4)	C31A—N24A—C34A—N26A	8.4 (4)
C29B—N24B—C34B—O8B	−2.5 (5)	C35A—N26A—C34A—O8A	−3.5 (5)
C31B—N24B—C34B—O8B	171.5 (3)	C32A—N26A—C34A—O8A	173.6 (3)
C29B—N24B—C34B—N26B	−179.5 (3)	C35A—N26A—C34A—N24A	177.9 (3)
C31B—N24B—C34B—N26B	−5.5 (4)	C32A—N26A—C34A—N24A	−4.9 (4)
C34B—N26B—C35B—N5B	99.8 (4)	C34A—N26A—C35A—N5A	−100.1 (4)
C32B—N26B—C35B—N5B	−77.7 (4)	C32A—N26A—C35A—N5A	83.1 (4)
C4B—N5B—C35B—N26B	−99.0 (4)	C4A—N5A—C35A—N26A	95.3 (4)
C1B—N5B—C35B—N26B	97.7 (4)	C1A—N5A—C35A—N26A	−93.5 (4)
C3B—N4B—C36B—N27B	106.5 (4)	C33A—N27A—C36A—N4A	106.2 (4)
C1B—N4B—C36B—N27B	−75.9 (4)	C32A—N27A—C36A—N4A	−91.7 (4)
C33B—N27B—C36B—N4B	−110.8 (4)	C3A—N4A—C36A—N27A	−107.0 (4)
C32B—N27B—C36B—N4B	97.2 (4)	C1A—N4A—C36A—N27A	80.4 (4)

### Pentaaqua(cucurbit[6]uril-κ2O,O')(nitrato-κ2O,O')praseodymium(III) dinitrate 9.56-hydrate Hydrogen-bond geometry (Å, º)

**Table:**

*D*—H···*A*	*D*—H	H···*A*	*D*···*A*	*D*—H···*A*
O1*WB*—H1*WA*···O3*B*	0.90	2.50	3.378 (4)	169
O2*WB*—H2*WA*···O11*W*^i^	0.87	1.85	2.674 (3)	157
O2*WB*—H2*WB*···O1*B*	0.87	2.07	2.865 (3)	150
O2*WB*—H2*WB*···O2*B*	0.87	2.46	2.894 (4)	111
O3*WB*—H3*WA*···O2*W*^i^	0.87	1.88	2.709 (4)	158
O3*WB*—H3*WB*···O4*B*	0.87	1.89	2.710 (3)	156
O4*WB*—H4*WA*···O1*W*^i^	0.87	2.01	2.729 (4)	139
O4*WB*—H4*WB*···O10*A*^ii^	0.87	2.48	3.211 (3)	142
O4*WB*—H4*WB*···O11*A*^ii^	0.87	2.60	3.206 (3)	127
O5*WB*—H5*WA*···O11*A*^ii^	0.87	2.08	2.798 (4)	140
O5*WB*—H5*WA*···O12*A*^ii^	0.87	2.44	3.018 (3)	124
O5*WB*—H5*WB*···O4*W*^i^	0.87	1.86	2.712 (4)	167
O1*WA*—H1*WC*···O2*A*	0.86	2.41	3.251 (4)	167
O1*WA*—H1*WD*···O7*W*^c^	0.85	1.99	2.749 (17)	148
O1*WA*—H1*WD*···O8*W*^a^	0.85	2.25	2.751 (9)	118
O2*WA*—H2*WC*···O1*A*	0.87	1.80	2.667 (3)	179
O2*WA*—H2*WD*···O19*W*^iii^	0.87	1.95	2.723 (4)	147
O3*WA*—H3*WC*···O4*A*	0.87	1.87	2.723 (3)	166
O3*WA*—H3*WD*···O16*W*	0.87	1.93	2.733 (4)	153
O4*WA*—H4*WC*···O10*B*^iv^	0.87	2.05	2.905 (3)	167
O4*WA*—H4*WD*···O14*W*^iii^	0.85	1.93	2.743 (4)	159
O5*WA*—H5*WC*···O19*W*^iii^	0.87	1.93	2.766 (5)	159
O5*WA*—H5*WD*···O9*B*^iv^	0.87	1.97	2.676 (3)	137
O15*W*—H15*A*···O3*A*	0.87	1.97	2.837 (4)	174
O15*W*—H15*B*···O16*B*	0.87	2.19	3.019 (5)	159
O15*W*—H15*B*···O18*B*	0.87	2.39	3.128 (5)	143
O15*W*—H15*B*···N2*B*	0.87	2.53	3.394 (5)	173
O1*W*—H1*WE*···O21*B*	0.87	1.92	2.768 (4)	165
O1*W*—H1*WF*···O16*A*	0.87	1.89	2.750 (4)	170
O17*W*^b^—H17*E*^b^···O2*A*^v^	0.87	2.07	2.913 (6)	163
O17*W*^b^—H17*F*^b^···O3*A*^v^	0.87	2.46	3.045 (9)	126
O3*W*—H3*WE*···O4*W*	0.87	1.86	2.734 (4)	177
O3*W*—H3*WF*···O8*A*^vi^	0.87	1.98	2.764 (3)	150
O2*W*—H2*WE*···O9*A*^vi^	0.87	2.41	3.047 (3)	131
O2*W*—H2*WE*···O3*W*	0.87	2.06	2.850 (4)	151
O2*W*—H2*WF*···O10*A*^vi^	0.87	1.96	2.822 (3)	173
O21*W*^a^—H21*A*^a^···O10*B*	0.88	2.41	3.181 (9)	145
O21*W*^a^—H21*B*^a^···O11*B*	0.90	2.36	3.213 (9)	157
O21*W*^a^—H21*B*^a^···O12*B*	0.90	2.60	3.127 (10)	118
O19*W*—H19*C*···O7*B*^i^	0.87	2.29	2.856 (4)	123
O19*W*—H19*C*···O8*B*^i^	0.87	2.28	2.939 (4)	132
O19*W*—H19*D*···O17*W*^b^	0.87	2.16	2.813 (7)	132
O19*W*—H19*D*···O18*W*^a^	0.87	1.70	2.54 (2)	160
O14*W*—H14*C*···O11*B*^i^	0.87	2.42	2.870 (4)	112
O14*W*—H14*C*···O12*B*^i^	0.87	2.21	3.024 (4)	156
O14*W*—H14*D*···O24*W*	0.85	2.25	2.913 (5)	135
O6*W*—H6*WA*···O9*A*	0.86 (2)	2.29 (2)	3.101 (5)	159 (4)
O6*W*—H6*WB*···O10*A*	0.87 (2)	2.31 (2)	3.117 (5)	155 (4)
O16*W*—H16*A*···O8*B*^iv^	0.87	2.17	3.014 (4)	163
O16*W*—H16*B*···O15*W*	0.87	1.93	2.723 (4)	152
O7*W*^c^—H7*WA*^c^···O6*W*	0.89	1.79	2.673 (17)	170
O4*W*—H4*WE*···O12*W*	0.87	1.89	2.744 (4)	166
O4*W*—H4*WF*···O7*A*^vi^	0.87	1.91	2.777 (3)	177
O8*W*^a^—H8*WA*^a^···O6*W*	0.87	1.93	2.737 (9)	153
O8*W*^a^—H8*WB*^a^···N21*A*	0.87	2.66	3.316 (9)	133
O18*W*^a^—H18*E*^a^···O2*A*^v^	0.87	2.08	2.94 (2)	171
O18*W*^a^—H18*F*^a^···O25*W*	0.87	2.35	2.79 (4)	111
O9*W*^b^—H9*WA*^b^···N19*A*	0.83	2.69	3.411 (10)	146
O9*W*^b^—H9*WB*^b^···O6*W*	0.86	1.94	2.805 (10)	177
O5*W*—H5*WE*···O14*W*	0.90	1.58	2.440 (11)	158
O5*W*—H5*WF*···O20*B*^i^	0.87	1.99	2.786 (12)	151
O10*W*—H10*A*···O4*B*^i^	0.87	2.21	2.981 (3)	148
O10*W*—H10*B*···O3*W*	0.87	1.83	2.697 (4)	175
O20*W*^b^—H20*C*^b^···O12*B*	0.87	2.00	2.86 (2)	167
O20*W*^b^—H20*D*^b^···O5*WA*^vii^	0.87	2.38	3.00 (2)	128
O20*W*^b^—H20*D*^b^···O19*W*^i^	0.87	2.37	3.20 (4)	160
O11*W*—H11*E*···O13*W*	0.87	1.91	2.749 (4)	161
O11*W*—H11*F*···O12*A*^vi^	0.87	1.90	2.755 (4)	168
O22*W*^a^—H22*A*^a^···O21*W*^a^	0.87	1.80	2.670 (11)	179
O22*W*^a^—H22*B*^a^···O1*WB*	0.87	1.93	2.763 (8)	161
O12*W*—H12*E*···O3*B*^i^	0.87	2.05	2.913 (4)	169
O12*W*—H12*F*···O2*B*^i^	0.87	2.15	2.918 (4)	147
O25*W*—H25*C*···O21*A*	0.87	2.02	2.872 (5)	166
O25*W*—H25*D*···O18*B*^viii^	0.87	2.09	2.953 (6)	170
O13*W*—H13*C*···O17*B*	0.87	2.03	2.884 (5)	165
O13*W*—H13*D*···O21*A*^ix^	0.87	1.96	2.800 (4)	163
O24*W*—H24*E*···O25*W*	0.87	1.93	2.790 (5)	171
O24*W*—H24*F*···O12*B*^i^	0.87	2.17	2.839 (4)	133

Symmetry codes: (i) −*x*+1, −*y*+1, −*z*+1; (ii) *x*+1/2, −*y*+3/2, *z*+1/2; (iii) −*x*+3/2, *y*+1/2, −*z*+1/2; (iv) *x*+1/2, −*y*+3/2, *z*−1/2; (v) −*x*+3/2, *y*−1/2, −*z*+1/2; (vi) −*x*+1/2, *y*−1/2, −*z*+1/2; (vii) *x*−1/2, −*y*+3/2, *z*+1/2; (viii) *x*+1/2, −*y*+1/2, *z*+1/2; (ix) *x*−1/2, −*y*+1/2, *z*−1/2.
